# An Overview of Autonomous Parking Systems: Strategies, Challenges, and Future Directions

**DOI:** 10.3390/s25144328

**Published:** 2025-07-10

**Authors:** Javier Santiago Olmos Medina, Jessica Gissella Maradey Lázaro, Anton Rassõlkin, Hernán González Acuña

**Affiliations:** 1 Program of Mechatronic Engineering, Universidad Autónoma de Bucaramanga, Bucaramanga 680003, Colombia; jolmos@unab.edu.co (J.S.O.M.); hgonzalez3@unab.edu.co (H.G.A.); 2 Department of Electrical Power Engineering and Mechatronics, Tallinn University of Technology, 19086 Tallinn, Estonia

**Keywords:** autonomous parking system, path planning, intelligent systems, environment recognition, end-to-end model-based system

## Abstract

Autonomous Parking Systems (APSs) are rapidly evolving, promising enhanced convenience, safety, and efficiency. This review critically examines the current strategies in perception, path planning, and vehicle control, alongside system-level aspects like integration, validation, and security. While significant progress has been made, particularly with the advent of deep learning and sophisticated sensor fusion, formidable challenges persist. This paper delves into the inherent trade-offs, such as balancing computational cost with real-time performance demands; unresolved foundational issues, including the verification of non-deterministic AI components; and the profound difficulty of ensuring robust real-world deployment across diverse and unpredictable conditions, ranging from cluttered urban canyons to poorly lit, ambiguously marked parking structures. We also explore the limitations of current technologies, the complexities of safety assurance in dynamic environments, the pervasive impact of cost considerations on system capabilities, and the critical, often underestimated, need for genuine user trust. Future research must address not only these technological gaps with innovative solutions but also the intricate socio-technical dimensions to realize the full potential of APS.

## 1. Introduction

### 1.1. Context and Motivation

Autonomous Parking Systems (APSs), encompassing functionalities ranging from Autonomous Parking Assist (APA) where the system handles steering while the driver controls speed, to fully Autonomous Valet Parking (AVP) where the vehicle parks itself without a driver inside, represent a significant area of advancement within automotive technology. These systems aim to alleviate the common stress and difficulty associated with parking maneuvers, particularly in constrained urban environments where precision is paramount and margins for error are minimal. The potential benefits are manifold, including enhanced driver convenience by automating a tedious task, improved spatial utilization in increasingly crowded parking areas through more precise and consistent parking, a reduction in minor collisions (dings, scrapes) often occurring during low-speed parking maneuvers, and contributing valuable technological building blocks to the broader development of fully autonomous vehicles (AV) [1]. The integration of Artificial Intelligence (AI), especially in perception and decision-making, has notably accelerated the development of these features, enabling more adaptive and seemingly intelligent behaviors [2]. Market forecasts reflect this potential, with the automotive AI market expected to grow significantly, driven partly by the demand for Advanced Driver Assistance Systems (ADASs) like APS.

**Figure 1:**
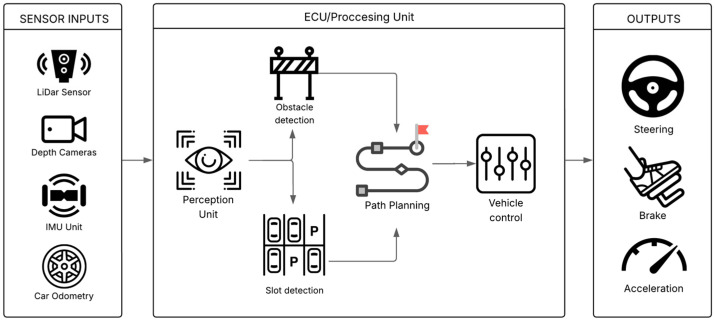
General architecture of a modular Autonomous Parking System (APS) illustrating core components and data flow.

However, despite considerable progress and the availability of various systems on the market, deploying APSs that can operate reliably and robustly across the full spectrum of real-world complexity remains a formidable challenge [3]. The transition from controlled demonstrations in well-defined test tracks to ubiquitous, robust real-world deployment in everyday parking scenarios is hampered not only by sheer environmental complexity, such as varying weather conditions, diverse parking lot layouts, and inconsistent human behaviors, but also by fundamental trade-offs. These include the delicate balance between system performance (e.g., speed and smoothness of parking) and the computational resources available on automotive-grade hardware, the rigorous demands of safety assurance versus the desire for rapid feature deployment, and the overarching influence of cost constraints on sensor suites and processing power. Furthermore, the persistent challenge of validating systems against the unpredictability of human behavior (e.g., pedestrians suddenly appearing, other drivers making unexpected maneuvers) and diverse, often poorly maintained, operational conditions adds layers of difficulty [4,5]. This review will critically examine these underlying issues, aiming to provide a nuanced understanding of the current state and future trajectory of APSs. Figure 1 illustrates the general architecture of such a system, outlining the primary components and the flow of information from sensor input to vehicle control output.

### 1.2. Recent Drivers of Progress

**Figure 2:**
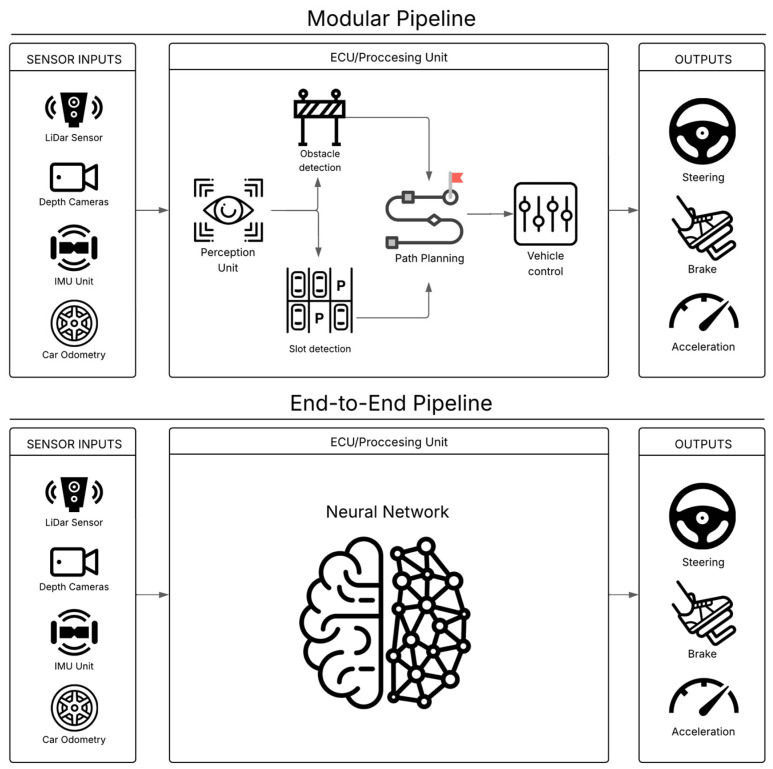
Comparison of modular pipeline and end-to-end (E2E) pipeline architectural paradigms for Autonomous Parking Systems.

Recent years have witnessed continued progress in APS research, fueled by several key technological drivers. Advances in sensor technologies, particularly the increasing resolution, field-of-view, and performance of automotive radar (e.g., 77 GHz and 79 GHz systems offering finer angular separation) [6] and LiDAR (light detection and ranging) providing denser point clouds for detailed 3D mapping [7,8], provide richer and more detailed environmental data. Simultaneously, more advanced fusion algorithms are emerging to seamlessly combine inputs from diverse sensor types—ultrasonic, camera, radar, and LiDAR, enhancing the system’s overall perception capabilities [9,10]. This fusion aims to overcome the limitations of individual sensors—for example, the poor performance of cameras in low light can be compensated for by the all-weather capabilities of radar, thereby improving overall perception accuracy and robustness against environmental uncertainties. Deep learning (DL) has become the dominant paradigm for perception tasks such as parking slot detection, free-space estimation, and obstacle identification [3,8,11], offering superior performance compared to traditional computer vision (CV) methods, especially in handling vast variations in lighting, weather, and object appearances where rule-based systems falter. Furthermore, research continues to yield more advanced path planning algorithms capable of generating smooth, kinematically feasible trajectories in cluttered and confined spaces, taking into account vehicle dynamics and passenger comfort [12,13,14]. Moreover, these refined vehicle control strategies enable precise and comfortable maneuver execution, minimizing jerky movements and ensuring accurate path tracking [15,16]. Therefore, these advancements collectively push the capabilities of APSs towards greater autonomy, reliability, and a more seamless user experience. These systems are generally designed using one of two primary architectural paradigms, the traditional modular pipeline or the more recent end-to-end approach, as compared in Figure 2.

## 2. Perception Methods for Parking Environments

Accurate and robust perception of the surrounding environment is fundamental for any APS. Research conducted in recent years has focused on enhancing sensor capabilities, refining fusion techniques, leveraging deep learning for detection tasks, and critically, improving performance under the challenging and diverse conditions encountered in real-world parking scenarios [3,17].

### 2.1. Sensor Technologies and Fusion

This section examines the specific sensor technologies utilized in Advanced Parking Systems (APSs), as well as the data fusion strategies applied to integrate their outputs and achieve a comprehensive understanding of the parking environment.

#### 2.1.1. Critical Analysis of Sensor Technologies

Table 1 provides a critical comparative analysis of key individual sensor technologies relevant to the development and deployment of Autonomous Parking Systems (APSs). Indeed, it aims to synthesize current knowledge, highlighting not only reported strengths but also inherent weaknesses, practical limitations, specific fusion challenges, and crucial cost–performance trade-offs.

#### 2.1.2. Critical Analysis of Sensor Fusion Approaches

Table 2 reports a critical comparative analysis of key sensor fusion approaches relevant to APS development and highlights strengths, weaknesses, limitations, fusion challenges, and cost–performance trade-offs.

Modern APSs continue to rely on a suite of sensors, typically including ultrasonic sensors, cameras, radar, and increasingly, LiDAR, to capture comprehensive information about the vehicle’s surroundings. Ultrasonic sensors remain prevalent for short-range distance measurements, crucial for final parking maneuvers and detecting low-lying curbs. Cameras provide rich visual information, which is essential for detecting parking lines, reading textual information on signs, and classifying objects [9]. Radar offers robustness in adverse weather conditions (rain, snow, fog) and provides direct velocity measurements of moving objects [6]. LiDAR provides precise 3D point clouds, enabling detailed environmental mapping, accurate object dimensioning, and improved localization [7,51]. A notable trend is the push towards higher-frequency radar systems (e.g., 77 GHz and 79 GHz), which offer improved resolution and object separation capabilities compared to traditional automotive radars, enabling finer environmental details to be captured, such as the delineation between closely parked vehicles [6,62], enabling finer environmental details to be captured, such as the delineation between closely parked vehicles. Indeed, the maturation of 4D imaging radar technology is evidenced by commercially available systems that provide dense point clouds detailing range, azimuth, elevation, and velocity, offering robust perception data essential for advanced APS functions [63].

However, the inherent limitations of individual sensors introduce critical challenges that may propagate throughout the entire APS workflow. For instance, while cameras provide rich visual data, their sensitivity to illumination changes (e.g., sudden glare from the sun, deep shadows in underground garages, rapid transitions from bright outdoor to dim indoor lighting) and adverse weather (e.g., rain droplets or mud obscuring lenses, heavy fog reducing visibility to near zero) can severely degrade performance [9,17,64]. So, a camera might fail to detect faded parking lines on a wet surface or misinterpret reflections as obstacles. Current mitigation strategies, such as High Dynamic Range (HDR) imaging and infrared (IR) illumination, may prove insufficient under all-weather and variable lighting conditions, particularly in the context of cost-sensitive mass-market vehicles, where the integration of high-end, expensive sensors is often not feasible. The practical consequences of sensor performance degradation can vary widely, ranging from incorrect parking space detection and failed parking attempts to the inability to identify small obstacles, such as animals or debris, thereby directly impacting both safety and system reliability.

**Figure 3:**
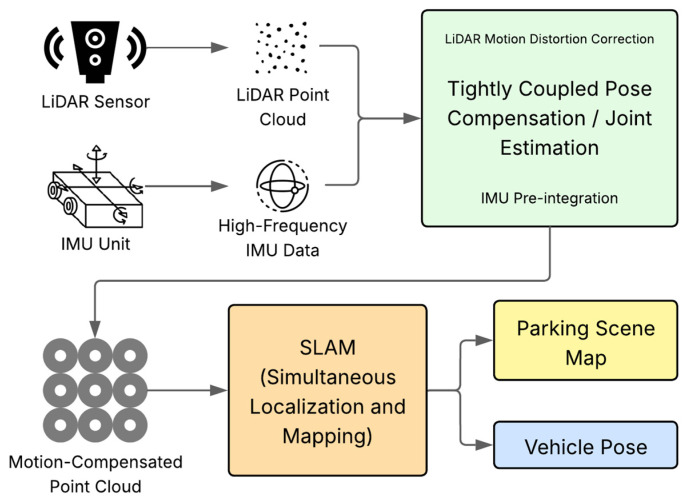
Architecture for tightly coupled LiDAR-IMU fusion, demonstrating the process from sensor input to motion-compensated point cloud generation and subsequent SLAM for map creation and vehicle pose estimation.

Sensor fusion is indispensable for creating a perception system that is more reliable and accurate than the sum of its parts, mitigating these inherent weaknesses by leveraging the complementary strengths of different sensor types [9,10,17,64]. Some authors explore various fusion strategies, ranging from geometry-based approaches that analyze patterns from fused multi-sensor data [10] to more complex, tightly coupled fusions of LiDAR and Inertial Measurement Unit (IMU) data for improved pose estimation and robust point cloud mapping, even during vehicle motion [8,65], as illustrated in Figure 3. Bird’s Eye View (BEV) fusion frameworks, which project sensor data onto a common top-down plane, are also gaining traction for their intuitive representation and ability to integrate information from multiple cameras and other sensors. Moreover, sensor fusion is intended to enhance the robustness of perception systems. However, the inherent complexity and current limitations of fusion algorithms introduce significant, often underacknowledged, challenges [1,17]. A critical question is the following: What are the inherent failure modes of current fusion algorithms? For example, how are inconsistencies between sensor modalities resolved, particularly in scenarios where radar detects an object that a camera fails to register due to occlusion or variations in material reflectivity? Similar challenges arise when a sensor provides clearly erroneous data as a result of malfunction, signal interference (e.g., radar jamming in dense environments), or specific environmental conditions, such as ultrasonic sensors generating ‘ghost’ readings from highly reflective surfaces. Moreover, does the reliance on sensor fusion introduce additional points of failure, including errors from miscalibration or temporal desynchronization, which could potentially lead to misaligned datasets? In conclusion, to what extent does the computational overhead incurred by integrating high-bandwidth sensor data streams induce latency levels that compromise the timeliness and reliability of decision-making processes in real-time, safety-critical applications? Furthermore, maintaining accurate calibration and temporal synchronization between diverse sensors in harsh automotive environments (subject to constant vibrations, wide temperature extremes, and potential physical damage) is a persistent operational challenge. Sensor drift over time, if not continuously monitored and corrected, can significantly degrade fusion reliability, leading to a gradual or sudden decline in perception performance.

Contemporary research embodies a pragmatic paradigm that meticulously reconciles technological innovation with prevailing market imperatives and rigorous cost-efficiency criteria. While advanced techniques, such as tightly coupled LiDAR-IMU fusion [8,65], push performance boundaries for high-end systems, significant effort is also directed towards enhancing the capabilities and robustness of lower-cost sensor configurations, often relying heavily on vision and ultrasonic sensors. This leads to a critical analysis point: while optimizing lower-cost sensor configurations is essential for increasing accessibility and market adoption of APS technology, it is critical to assess the quantifiable performance limits and safety trade-offs of these budget-conscious systems compared to high-end alternatives, especially under challenging conditions such as heavy snowfall or in complex environments like densely packed or irregularly shaped parking spaces. Is there a definable threshold in sensor quality or configuration below which reliable and safe APS operation becomes unfeasible, regardless of advances in algorithmic processing or fusion strategies? This trade-off between cost and performance, especially concerning safety-critical functions where failure can have severe consequences, requires more transparent evaluation and clear communication of system limitations to end-users.

### 2.2. Deep Learning for Parking Space and Obstacle Detection

**Figure 4:**
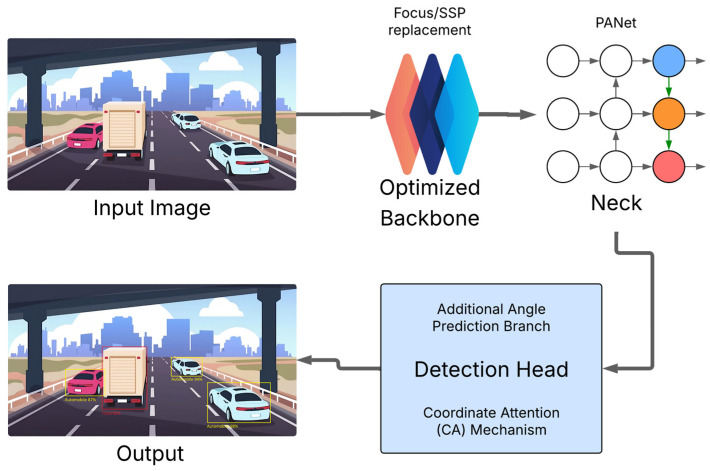
Enhanced YOLOv5-OBB model architecture for oriented bounding box detection in parking scenarios, featuring an optimized backbone, PANet neck, and a detection head with an additional angle prediction branch and Coordinate Attention (CA) mechanism.

DL, particularly using Convolutional Neural Networks (CNNs), has become the standard approach for parking space detection, largely superseding traditional computer vision techniques that relied on handcrafted features like edge detection or Hough transforms [3,9]. DL methods demonstrate superior performance, especially in handling vast variations in lighting, weather conditions, diverse parking space markings (or lack thereof), and varied appearances of obstacles, where traditional methods often falter due to their rigidity [11,66,67]. Architectures like YOLO (You Only Look Once) and its variants are frequently adapted for their balance of speed and accuracy in detecting parking slots as bounding boxes [3,68], with enhancements such as oriented bounding boxes and attention mechanisms being developed to improve performance in specific parking scenarios (Figure 4). Recent work includes improvements that focus not only on accuracy (e.g., mean Average Precision, mAP) but also on inference speed (Frames Per Second, FPS) and reducing model size for deployment on resource-constrained embedded automotive hardware [3]. Segmentation-based models (e.g., Mask R-CNN) [69], which classify each pixel, are also employed for more precise delineation of parking spaces and drivable areas. Hybrid DL-computer vision approaches, which might use DL for initial region proposal and traditional techniques for refinement [11], are also explored to balance performance and computational load.

While metrics like mAP and FPS demonstrate progress in academic benchmarks and controlled tests [3,11], a critical question is the following: How well do these correlate with real-world robustness and safety, especially concerning rare but critical ‘edge cases’ (e.g., an unusually shaped obstacle, a parking spot partially obscured by overhanging foliage, or ambiguous markings during construction) not well-represented in training datasets [3]? And, do current benchmarks, often composed of curated and relatively clean data, adequately capture a model’s ability to generalize to entirely novel parking environments, different geographical regions with unique parking conventions, or unforeseen obstacle types? The focus on benchmark performance might not fully translate to reliability in the unpredictable and often messy real world, potentially leading to a gap between perceived and actual system capability.

The superior performance of DL methods often comes at the cost of interpretability [2,66]. This “black box” nature, where the internal reasoning of the network is opaque even to its developers, poses a significant challenge for safety validation and debugging. How can we ensure the reliability and predictability of DL-based perception when the decision-making process is not transparent, and what are the implications for certifying such systems as safe according to stringent automotive safety standards like ISO 26262 [70]? To mitigate the risks associated with this lack of transparency, the research community is actively pursuing several key strategies aimed at rendering AI-based systems more scrutable and trustworthy. Methodologies such as:

Explainable AI (XAI) seeks to provide insights into the internal reasoning of a network, for instance, by generating visual attribution maps that highlight which input features most influenced a particular decision. Another prominent approach is the development of hybrid models, which strategically combine the high-performance capabilities of deep learning for perception tasks with the formal verifiability of traditional, model-based algorithms for safety-critical logic and decision-making. Furthermore, uncertainty estimation is a critical technique wherein the model outputs not only a prediction but also a corresponding confidence level. This allows the system to recognize when it is operating in a low-confidence or out-of-distribution scenario, enabling it to trigger fallback maneuvers or request human intervention, thereby providing a crucial safety margin.

Furthermore, DL’s heavy reliance on large datasets introduces concerns about data dependency and bias [3,66]. Given DL’s heavy reliance on large datasets, how do biases present in these datasets (e.g., overrepresentation of certain parking types like perpendicular slots in well-lit conditions, specific weather conditions common to the data collection region, or particular geographic locations with uniform parking infrastructure) affect the fairness and robustness of deployed APS? What are the risks if a system performs exceptionally well in one region or under specific conditions but poorly in another due to dataset bias, potentially leading to inequitable performance or unexpected failures when encountering underrepresented scenarios? This can lead to systems that are not equitable or universally reliable, for instance, performing worse in regions with non-standard parking markings or in weather conditions rare in the training data.

To systematically address these challenges of data dependency, bias, and the critical need to cover rare but consequential ‘edge cases,’ the field employs a multi-pronged data strategy. A foundational technique is **data augmentation**, where existing data is algorithmically manipulated by altering lighting, applying simulated weather effects, or adding noise to create a wider variety of training examples without new data collection. For scenarios that are too dangerous, costly, or infrequent to capture in reality, **synthetic data generation** using high-fidelity automotive simulators like CARLA has become indispensable; it allows for the creation of vast, perfectly annotated datasets encompassing a nearly infinite variety of environmental conditions and event sequences. Additionally, more advanced paradigms are employed: **active learning** optimizes the expensive process of human annotation by having the model itself flag uncertain or novel scenarios that it cannot confidently interpret, thereby focusing expert review on the most valuable learning opportunities. To overcome privacy barriers and geographic bias, **federated learning** allows a central model to learn from the real-world experiences of a globally distributed fleet of vehicles without the raw sensor data ever leaving the individual car. Finally, these methods are complemented by **targeted real-world sampling**, a strategic approach where data collection fleets are specifically deployed to underrepresented domains such as regions with non-standard infrastructure or during specific adverse weather events to consciously fill known coverage gaps and mitigate inherent biases in the training data.

A significant practical constraint is computational efficiency for automotive-grade embedded systems, which have limited processing power and strict thermal envelopes [2,3]. The drive for computational efficiency often leads to model compression techniques (e.g., pruning, quantization) or the use of shallower, less complex neural networks. Critically, what is the quantifiable impact of such optimizations on the model’s ability to detect subtle features (e.g., a faintly visible parking line, a small, low-contrast obstacle), handle occlusions effectively, or maintain performance in challenging, noisy conditions? Is there a point where efficiency gains, driven by cost or power limitations, lead to an unacceptable degradation in safety-critical perception tasks, potentially missing a critical obstacle or misjudging a parking space boundary? This trade-off requires careful, systematic balancing and rigorous testing to ensure that safety is not unduly compromised for the sake of speed or cost.

### 2.3. Achieving Robustness in Diverse Conditions

A persistent challenge for APS perception systems is maintaining reliable performance across the wide range of conditions encountered in real-world parking environments [3,17,64]. These include variations in illumination (bright sunlight causing lens flare, deep shadows obscuring details, rapidly changing light at dusk/dawn, various types of artificial indoor lighting with different spectral properties), adverse weather (rain, snow, fog, sleet, hail) [64,71], occlusions (by other vehicles, pedestrians, shopping carts, structural pillars), and inconsistencies or degradation in parking space markings (faded lines, non-standard colors or types, temporary markings, or complete absence of markings in informal parking areas).

Training DL models on large, diverse datasets that encompass a wide variety of these conditions is a common and essential strategy [11]. Sensor fusion inherently contributes to robustness by providing complementary information; for instance, radar’s performance is less affected by poor lighting or precipitation compared to cameras [6,62], while LiDAR can penetrate some level of fog or rain better than cameras.

The scarcity of comprehensive public datasets is a recurring theme and a significant impediment to progress [7,72]. However, it is crucial to analyze why creating such datasets is profoundly difficult. Beyond sheer volume and the cost of collection and annotation, the challenge lies in capturing the long tail of rare events—those infrequent but potentially critical scenarios that a system must handle safely. This includes unusual obstacle types, peculiar parking geometries, complex interactions with other road users, and diverse sensor noise profiles under extreme conditions. Furthermore, capturing the subtle environmental cues that human drivers intuitively process (e.g., slight changes in road texture indicating a parking boundary, or the behavior of other drivers suggesting an imminent maneuver) is exceptionally hard to codify and include in datasets. Are current data collection (e.g., fleet vehicles) and annotation methods (manual and semi-automated) scalable or sophisticated enough to address this fundamental bottleneck effectively and economically? This data scarcity significantly hampers the development and rigorous evaluation of robust perception algorithms, making it difficult to benchmark different approaches fairly [7,72], consistently reproduce research findings, and train models that generalize well to unseen environments.

While training on diverse datasets [11,71] and employing sensor fusion are common strategies, to what extent do these approaches guarantee robustness against truly ‘out-of-distribution’ scenarios (i.e., situations fundamentally different from anything in the training data) or compound failures (e.g., a primary sensor malfunction occurring simultaneously with adverse weather conditions)? Current strategies often represent incremental improvements that may still leave significant performance gaps in extreme or unforeseen conditions, rather than providing a comprehensive, verifiable solution to the robustness problem. They may make the system more robust, but not necessarily sufficiently robust for all conceivable real-world encounters.

Table 3 provides a comparative summary of several recent deep learning-based methods, highlighting their architectural innovations, the datasets used for evaluation, and key performance metrics.

## 3. Path Planning Algorithms for APS

Generating a safe, feasible, and efficient path from the vehicle’s current position to the target parking spot is a core function of APSs, requiring a delicate balance between geometric constraints, vehicle dynamics, safety margins, and passenger comfort [74]. Research in recent years has advanced various planning paradigms, often combining techniques to leverage their respective strengths, particularly for navigating complex and constrained parking environments.

### 3.1. Optimization and Control-Based Planning

Formulating parking trajectory generation as an Optimal Control Problem (OCP) is a powerful approach that allows for the direct minimization of objectives like parking time, path length, or control effort (e.g., minimizing steering changes) while simultaneously satisfying vehicle dynamic constraints (e.g., maximum curvature, velocity limits) and collision avoidance criteria [12]. The general form of an OCP can be expressed as:

minimize the cost functional J:
  (1) $$J=\phi (x(t_{f}),t_{f})+\int_{t_{0}}^{t_{f}}\;L(x(t),u(t),t)dt,$$
  subject to system dynamics:
  (2) $$\dot{x}(t)=f(x(t),u(t),t),$$
  initial conditions:
  (3) $$x(t_{0})=x_{0},$$
  and path/terminal constraints:
  (4) ψ(x(t),u(t))≤0,
  (5) $$\chi (x(t_{f}))=0.$$
  where J is the performance index, $\phi (x(t_{f}),t_{f})$ is the terminal cost, L(x(t),u(t),t) is the instantaneous cost (Lagrangian), x(t) is the state vector, u(t) is the control input vector, f(x(t),u(t),t) represents the vehicle dynamics, $\psi (x\left(t\right)\;and\;u(t))$ are path constraints (e.g., actuator limits, obstacle avoidance), and $\chi (x(t_{f}))$ are terminal constraints (e.g., reaching the desired parking pose).

Recent work has demonstrated the use of indirect optimal control methods, solved efficiently using specialized numerical tools, which can achieve computation times suitable for near real-time application, even with fine discretization needed for navigating close to obstacles [12]. Numerical optimization techniques, such as Sequential Quadratic Programming (SQP), are also employed, often in conjunction with other methods. For instance, SQP can optimize the control points of Bézier curves used for path generation, minimizing a cost function that includes terms for path smoothness (curvature, jerk), safety (based on an Artificial Potential Field representing obstacles), and proximity to the target pose [16]. Model Predictive Control (MPC) is another relevant technique, used either directly for planning over a receding horizon [75] or, more commonly in the recent literature, for trajectory optimization and tracking [13]. These methods can produce high-quality, smooth, and dynamically feasible trajectories, but they often face computational challenges, especially when dealing with complex environments featuring many obstacles and non-convex constraints. Finding a globally optimal solution can be time-consuming, and the performance heavily depends on the problem formulation and the availability of a good initial guess or starting point for the optimization process, which if poor, can lead to local minima or slow convergence [12,74]. This dependency often leads researchers to adopt hierarchical or hybrid approaches [13,14,76].

### 3.2. Search and Sampling-Based Planning

Graph search and random sampling algorithms remain popular for exploring the configuration space (the space of all possible vehicle positions and orientations) and finding kinematically feasible paths, especially in complex or initially unknown environments [74]. The A* algorithm, a cornerstone of graph search, evaluates nodes using the function:

(6) f(n)=g(n)+h(n),

where f(n) is the estimated total cost of the path from the start node to the goal node through the node n, g(n) is the actual cost from the start node to the node n, and h(n) is the heuristic estimate of the cost from the node n to the goal.

The Hybrid A* algorithm, which combines the grid-based search of A* with continuous state expansion respecting vehicle kinematics (e.g., Reeds–Shepp curves), is particularly prevalent [13,14]. Recent enhancements focus on improving its efficiency and the quality of the generated path, for example, through adaptive node expansion strategies that adjust the search step length and angle based on environmental complexity and proximity to the goal [14]. Heuristic functions are also being improved by incorporating information from simpler, faster global planners to better guide the Hybrid A* search.

Rapidly exploring Random Tree (RRT) algorithms and their variants (RRT*, Bi-RRT*, RRT-Connect) are also widely used due to their ability to quickly explore high-dimensional spaces and find paths without explicit free-space modeling [77,78]. Improvements aim to address the inherent randomness and sometimes suboptimal (e.g., overly long or jerky) paths generated by basic RRT. While effective at finding an initial, collision-free, and kinematically feasible path, the resulting path often lacks the smoothness (e.g., continuous curvature) and optimality required for comfortable and precise execution by a real vehicle [14,74].

**Figure 5:**
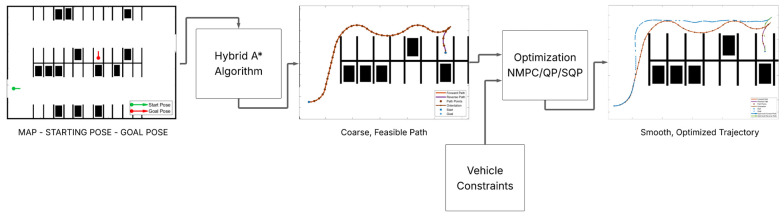
Hierarchical path planning framework for APSs, showing an initial path generated by the Hybrid A* algorithm refined by an Optimization stage (e.g., NMPC (Nonlinear Model Predictive Control)/QP/SQP) considering vehicle constraints.

The prevalence of hybrid approaches, which combine a search/sampling-based algorithm for generating an initial path with an optimization-based method for subsequent refinement (as shown in Figure 5), strongly suggests that neither paradigm is sufficient in isolation for the multifaceted complexities of APSs [13,14,76]. Critically, does this reliance on multi-stage planning introduce cumulative errors, where small inaccuracies in the initial path are magnified during refinement? Does it increase computational overheads due to sequential processing, or create new challenges in ensuring global optimality or even consistent, predictable behavior across different scenarios? For example, a suboptimal initial path might lead the refiner into a local minimum from which it cannot escape to find a better overall solution.

Delving into the trade-offs, optimization-based methods [12,16], while capable of producing smooth, dynamically feasible paths, often struggle with non-convex environments leading to local minima (becoming stuck in a suboptimal solution), and their computational cost can be prohibitive for real-time replanning in dynamic scenarios where obstacles might move or new information becomes available. The complexity of formulating all relevant constraints accurately can also be a hurdle. Conversely, while sampling-based planners like RRT* [77] can navigate complex spaces and are probabilistically complete (meaning they will find a path if one exists, given enough time), the paths generated are often suboptimal in terms of length or smoothness, requiring significant post-smoothing. This post-smoothing process may itself re-introduce constraint violations (e.g., by smoothing a corner too tightly, making it kinematically infeasible) or cause the path to deviate significantly from the initial ‘safe’ path found by the sampler.

What are the fundamental limits of each approach such as the curse of dimensionality for grid-based search or the difficulty of incorporating complex cost functions into sampling that necessitate these hybrid solutions, and what compromises are being made in terms of path quality, computational time, guarantee of finding a solution, and predictability of the final trajectory?

Moreover, although many planners effectively manage static obstacles with well-defined geometries, the real-time, robust, and efficient navigation around unpredictable dynamic entities such as pedestrians abruptly crossing, vehicles unexpectedly reversing, or freely moving shopping carts within the constrained spaces and limited visibility typical of parking lots, continues to represent a significant and largely unresolved challenge [16,79]. How do current approaches balance proactive avoidance (maintaining safe distances) with maintaining progress towards the goal without resorting to overly conservative (e.g., stopping frequently) or jerky maneuvers that could be disconcerting to passengers or other road users? The ability to predict the intent and future motion of these dynamic actors is crucial yet exceedingly difficult.

### 3.3. Addressing Constraints and Complexities

A major focus of recent APS path planning research is tackling challenging real-world scenarios. This includes planning maneuvers in narrow or tightly constrained parking spots where clearance is minimal [12,13,76], operating in unstructured environments without clear markings or predefined layouts (e.g., gravel lots, informal street parking), and safely navigating environments with multiple static or dynamic obstacles [79,80]. Non-holonomic kinematic constraints (minimum turning radius, steering angle limits, vehicle dimensions) are explicitly considered during node expansion in algorithms like Hybrid A* [13,14] or are formulated as hard constraints within OCP or MPC frameworks [12]. A common representation used for vehicle kinematics is the bicycle model:

(7) $${\dot{x}}_{v}=vcos(\psi ),$$

(8) $${\dot{y}}_{v}=vsin(\psi ),$$

(9) $$\dot{\psi }=\frac{v}{L}tan(\delta ),$$

with constraints such as:

(10) $$|\delta |\leq \delta_{max},$$

(11) $$|\dot{\delta }|\leq {\dot{\delta }}_{max},$$

(12) $$R_{min}=\frac{L}{\left|tan(\delta_{max})\right|},$$

where $\left({\dot{x}}_{v},{\dot{y}}_{v}\right)$ are the vehicle’s longitudinal and lateral velocities, v It’s speed, ψ is the orientation (yaw) angle, δ is the steering angle of the front wheels, L is the wheelbase, $\delta_{max}$ and ${\dot{\delta }}_{max}$ are the maximum steering angle and rate, and $R_{min}$ is the minimum turning radius.

**Figure 6:**
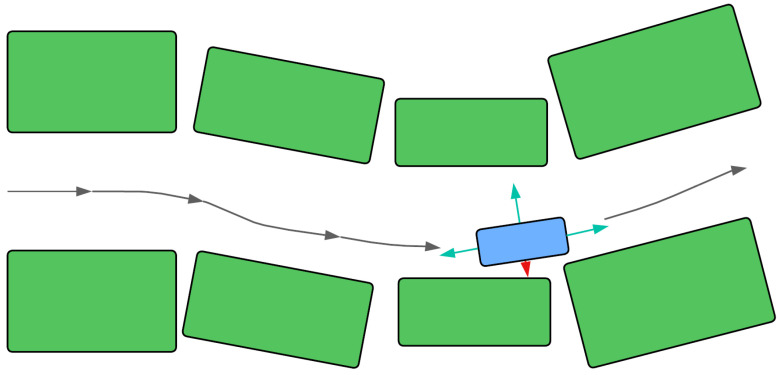
Conceptual illustration of the Improved Safe Travel Corridor (I-STC), which defines a collision-free rectangular region around the vehicle, oriented along its current heading, by expanding until it hits obstacles or a maximum distance. The green arrows represent the calculated safe clearance distances, while the red arrow indicates a dangerously close proximity.

Collision avoidance is handled through various means: smooth, differentiable penalty functions added to OCP cost functions that penalize proximity to obstacles [12]; repulsive forces generated by obstacles within an Artificial Potential Field (APF) framework [16,77]; or geometric constructions like the Improved Safe Travel Corridor (I-STC), illustrated conceptually in Figure 6, that define a collision-free corridor around an initial path, simplifying the constraints for subsequent optimization stages [76]. In APF methods, the total potential U(q) at a configuration q is often a sum of an attractive potential towards the goal $U_{att}(q)$ and a repulsive potential from obstacles $U_{rep}(q)$:

(13) $$U(q)=U_{att}(q)+U_{rep}(q),$$

an attractive potential can be:

(14) $$U_{att}(q)=\frac{1}{2}k_{att}\|q-q_{goal}{\|}^{2},$$

and a repulsive potential for an obstacle i is:

(15) $$U_{rep,i}(q)=\left\{\begin{array}{ll} \frac{1}{2}k_{rep}{\left(\frac{1}{\rho (q,obs_{i})}-\frac{1}{\rho_{0}}\right)}^{2} & \mathrm{if}\;\rho (q,obs_{i})\leq \rho_{0}\\ 0 & \mathrm{if}\;\rho (q,obs_{i})>\rho_{0} \end{array}\right.,$$

where $k_{att}$ and $k_{rep}$ are gain coefficients, $q_{goal}$ is the goal configuration, $\rho (q,obs_{i})$ is the minimum distance to obstacle i, and $\rho_{0}$ is its distance of influence. The vehicle then follows the negative gradient of this field: F(q)=−∇U(q).

Planners are also being developed to handle complex maneuvers involving multiple gear shifts (forward and reverse segments), often required for parking in tight parallel or perpendicular spots [12,81].

### 3.4. Path Smoothing and Refinement

Generating paths that are not only feasible and collision-free but also smooth and comfortable for passengers is crucial for practical APS. Raw paths from search or sampling algorithms often contain sharp turns, discontinuities in curvature, or unnecessary oscillations [14,74]. Therefore, post-processing or integrated smoothing techniques are commonly applied. Geometric curves known for their smoothness properties, such as Bézier curves (offering flexibility through control points) [16] and Clothoid curves (Euler spirals, whose curvature varies linearly with arc length, naturally representing steering transitions), are frequently used.

A cubic Bézier curve, for instance, is defined by four control points ($P_{0},P_{1},P_{2},P_{3}$) as:

(16) $$B(t)=P_{0}(1-t{)}^{3}+3P_{1}t(1-t{)}^{2}+3P_{2}t^{2}(1-t)+P_{3}t^{3},t\in [0,1],$$

Clothoid curves are characterized by their curvature κ varying linearly with arc length s:

(17) $$\kappa (s)=\kappa_{0}+\sigma s,$$

or, if starting with zero curvature, $\kappa (s)=s/A^{2}$, where A is the clothoid parameter and σ is the sharpness.

Optimization-based smoothing, utilizing techniques such as Quadratic Programming (QP) [14] or Sequential Quadratic Programming (SQP) [16], adjusts path points or curve parameters to minimize objectives related to curvature, jerk, and deviation from the original path.

The necessity of incorporating explicit path smoothing stages reveals a fundamental shortcoming in many initial path generation algorithms. These algorithms typically focus on quickly identifying any feasible path that satisfies basic constraints, often at the expense of path quality metrics such as smoothness, continuity, and minimal curvature. This prioritization results in trajectories that may be suboptimal for practical execution, necessitating subsequent refinement processes to enhance drivability, passenger comfort, and overall system performance. Does this post-processing step guarantee the maintenance of safety margins established by the initial planner, or could smoothing inadvertently move the path closer to obstacles, potentially reducing clearance below acceptable levels in an attempt to achieve a more aesthetically pleasing or comfortable trajectory? For example, smoothing a sharp turn might slightly cut a corner, bringing the vehicle closer to a parked car than the initial planner intended. Moreover, how can the trade-off between path smoothness—critical for passenger comfort and precise trackability by the vehicle’s control system—and path length or maneuver time—key factors for operational efficiency and user satisfaction—be rigorously quantified and systematically optimized, rather than relying on heuristic adjustments driven by developer intuition or limited empirical validation? The absence of standardized, objective metrics to evaluate and balance these competing criteria often results in inconsistent system performance and complicates comparative assessments of various path smoothing methodologies.

Table 4 provides a comparative overview of the diverse path planning strategies discussed, summarizing their key features, how they handle vehicle constraints, and their validation methods.

## 4. Vehicle Control Strategies

Once a suitable path is planned, the vehicle’s control system is responsible for executing the maneuver by accurately tracking the trajectory and managing the vehicle’s actuators (steering, throttle, brakes). Research in this area focuses on developing controllers that ensure precision, stability, smoothness, and robustness while respecting physical limitations and passenger comfort [83].

### 4.1. Advanced Trajectory Tracking Controllers

Several advanced control strategies have been investigated and applied to the APS problem:

- Model Predictive Control (MPC) remains a prominent technique due to its inherent ability to handle constraints (on states and inputs) explicitly and optimize control actions over a future prediction horizon [13,75]. It can anticipate future path requirements and adjust current inputs accordingly, leading to smoother control [4]. A common discrete-time cost function for MPC in trajectory tracking is:

(18) $$J(x_{k},U_{k})=\sum_{i=0}^{N_{p}-1}\;(\|x_{k+i|k}-x_{ref,k+i}{\|}_{Q}^{2}+\|u_{k+i|k}-u_{ref,k+i}{\|}_{R}^{2})+\|x_{k+N_{p}|k}-x_{ref,k+N_{p}}{\|}_{P}^{2}$$

Subject to the discretized system dynamics $x_{k+i+1|k}=f_{d}(x_{k+i|k},u_{k+i|k})$ and constraints on states x and inputs u. Here, $N_{p}$ is the prediction horizon, $x_{ref}$ and $u_{ref}$ are reference states and inputs, and Q,R,P are weighting matrices.

**Figure 7:**
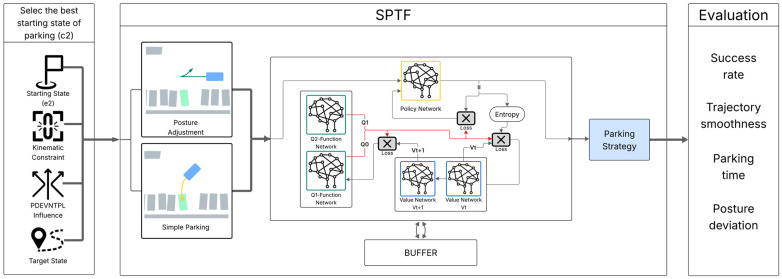
Architecture of the Segmented Parking Training Framework (SPTF) employing a Soft Actor–Critic (SAC) agent, illustrating inputs, training stages, network components, and evaluation metrics [15].

- Reinforcement Learning (RL) techniques, which are fundamentally based on agents learning optimal behavior through trial-and-error interactions with an environment to maximize a cumulative reward signal, are gaining traction for learning control policies directly from these interactions (or, more commonly, a simulation) [84,85]. Algorithms like Soft Actor-Critic (SAC), whose architecture is detailed in Figure 7, have been used to train parking strategies that explicitly balance multiple objectives, including safety, comfort (e.g., minimizing jerk), efficiency (e.g., minimizing time), and accuracy (e.g., final pose error) [15,82]. SAC maximizes an entropy-regularized objective:

(19) $$J(\pi )=\sum_{t=0}^{T}\;E_{(s_{t},a_{t})∼\rho_{\pi }}\left[r(s_{t},a_{t})+\alpha H(\pi (\cdot |s_{t}))\right]$$

where $r(s_{t},a_{t})$ is the reward, α is a temperature parameter, and $H(\pi (\cdot |s_{t}))$ is the policy entropy, encouraging exploration. Hybrid RL approaches, combining rule-based planners with learned components [82,86], are also being explored to leverage the strengths of both paradigms.

The increasing application of Deep Reinforcement Learning (DRL), particularly model-free methods like Soft Actor–Critic, represents a significant trend in vehicle control for autonomous parking. Unlike traditional controllers that rigidly follow pre-computed geometric paths, DRL agents can learn complex, adaptive control policies directly from interaction. This approach is particularly well-suited to the non-linear dynamics and multi-objective nature of parking, where the system must continuously balance safety margins, passenger comfort (e.g., minimizing jerk), maneuver efficiency, and final placement accuracy. By defining these objectives within a reward function, DRL can discover novel, high-performance parking strategies that are difficult to hand-engineer. However, as noted in the critical evaluation of these strategies, this power comes with significant challenges in formal verification, sim-to-real transfer, and ensuring robust performance against out-of-distribution scenarios, which remains a primary focus of ongoing research.

- Neural Network (NN)-Based Controllers; Beyond standard RL algorithms, researchers are developing bespoke NN-based controllers. One example is a pseudo-neural network (PNN) steering controller designed with a physics-driven structure, using feedforward components and feedback terms, trained via supervised learning on data from high-fidelity simulations [12].

Reinforcement Learning (RL), particularly SAC [15,82], shows promise for optimizing complex objectives and adapting to nuanced situations not easily captured by traditional models. However, its application in safety-critical APS faces significant hurdles: the ‘black box’ nature of learned policies makes formal verification of their safety and stability exceptionally difficult [2]; ensuring safety during the trial-and-error learning phase (especially if conducted in real-world interactions, which is rarely feasible) is problematic and typically relies on carefully designed simulators and reward functions; and the sim-to-real gap can lead to unpredictable or degraded real-world performance if the simulation doesn’t perfectly capture all aspects of vehicle dynamics and environmental interactions [15,82,86]. How are researchers addressing these fundamental safety and reliability concerns beyond demonstrating high reward scores in simulated environments? Are current validation methods for RL policies, often based on empirical testing over a limited set of scenarios, sufficient for safety certification in a domain where unexpected failures can have severe consequences?

MPC’s effectiveness is intrinsically linked to the fidelity of the vehicle model it uses for prediction [4,83]. In real-world parking scenarios, unmodelled dynamics (e.g., tire slip on varied surfaces like ice, wet leaves, or loose gravel; suspension changes due to passenger load or uneven weight distribution; aerodynamic effects at higher speeds, though less relevant for parking; or even subtle changes in tire pressure) or sudden external disturbances (e.g., wind gusts in exposed parking lots) can degrade MPC performance, leading to tracking errors or suboptimal control [13]. How robust are current MPC strategies to such model-reality mismatches, and what are the adaptation mechanisms in place (e.g., adaptive MPC, robust MPC formulations)? Over-reliance on an imperfect model can lead to suboptimal or even unsafe control actions if the controller’s predictions diverge significantly from reality.

Similar to DL in perception, NN-based controllers [12], whether learned through RL or supervised methods, raise profound questions about robustness to out-of-distribution states (i.e., situations or sensor inputs significantly different from those encountered during training) and the formal verification of their stability and safety envelopes across the entire operational domain. Without rigorous verification methods that can provide mathematical guarantees of safe behavior, their deployment in safety-critical systems like APSs remains contentious and reliant on extensive, but inevitably incomplete, empirical validation.

### 4.2. Ensuring Precision, Stability, and Handling Constraints

The primary goal of the trajectory tracking controller is to minimize the error between the vehicle’s actual position and orientation and the desired path. High precision is critical, especially in tight parking spots where millimeters can make the difference between a successful park and a collision [87]. Stability during the maneuver is paramount; controllers must ensure the vehicle does not exhibit undesirable oscillations or unstable behavior [83]. Furthermore, they must operate within the physical limitations of the vehicle’s actuators, respecting constraints on steering angle, steering rate, acceleration, and braking capabilities. Passenger comfort, often related to minimizing jerk (the rate of change of acceleration) [15,16], is another crucial consideration, as overly aggressive or jerky maneuvers can be unsettling.

Achieving high precision is often framed as a primary goal, and some research reports impressively low tracking errors [87]. However, is there a point where striving for sub-centimeter precision yields diminishing returns in practical parking success, especially if it comes at the cost of increased maneuver time (due to slower, more cautious movements), higher computational load (for more complex controllers), or overly sensitive control actions that might reduce passenger comfort or perceived safety (e.g., frequent, tiny steering adjustments)? How is ‘sufficient precision’ defined and justified in the context of overall system performance, user acceptance, and varying environmental conditions (e.g., is sub-centimeter precision truly necessary or even achievable on a highly uneven surface)? An overemphasis on precision in one aspect might neglect other important operational qualities or lead to an overly complex and sensitive system.

### 4.3. Critical Evaluation of Vehicle Control Strategies in Autonomous Parking Systems (APS)

#### Introduction to the Comparative Table

The following table, “Table 5: Critical Evaluation of Vehicle Control Strategies in Autonomous Parking Systems (APSs),” provides a synthesized and critical overview of prominent vehicle control strategies employed in Autonomous Parking Systems.

## 5. Comparative Analysis of Recent APS Research and System Developments

### 5.1. Introduction to Comparative Framework

To synthesize the rapid advancements in Autonomous Parking Systems, this section provides a comparative analysis of pivotal research studies and system developments. The focus is primarily on contributions from 2023 to 2025, supplemented by earlier works that represent critical foundational advances directly influencing current APS technology.

Grounding the advanced research presented here in practical reality, it is essential to analyze the state of Autonomous Parking Systems (APSs) currently available in the commercial market. These systems are not monolithic; rather, they exist on a spectrum of automation, a distinction this review makes between Automatic Parking Assist (APA) and fully Autonomous Valet Parking (AVP). The most common systems deployed by major manufacturers function at the APA level, providing steering control while the human driver remains responsible for managing the vehicle’s speed and monitoring the environment.

Moving toward greater autonomy, well-known systems like Tesla’s Autopark automate the entire steering and speed control sequence for parallel or perpendicular parking while the driver is inside. Tesla’s more advanced Smart Summon feature exemplifies an AVP-like capability, allowing a user to call the vehicle from its parking spot to their location remotely. Similarly, Hyundai’s Remote Smart Parking Assist (RSPA) provides remote control for maneuvering into tight spaces. While impressive, the real-world performance of these systems is often constrained; they function most reliably in well-marked lots with ample space and can exhibit hesitant or unpredictable behavior in crowded, dynamic environments. This performance envelope is a direct consequence of their reliance on a sensor suite composed primarily of cameras and ultrasonic sensors, which can struggle with the edge cases and adverse conditions detailed throughout this review. This highlights the significant gap that advanced research, focusing on robust multi-modal fusion with technologies like LiDAR and 4D radar, aims to close.

### 5.2. Comparative Table of Pivotal APS Studies and Systems

Table 6 summarizes selected recent and critical studies and system developments in APSs.

### 5.3. Analysis of Overarching Trends and Innovations

The comparative analysis presented in the previous section reveals several dominant trends and significant innovations in the APS landscape between 2023 and 2025. There is a clear and accelerating convergence towards AI-centric solutions, particularly Deep Reinforcement Learning (DRL) and end-to-end learning models, for tackling the core planning and control logic of autonomous parking. This indicates a strategic shift away from purely geometric or rigidly rule-based systems, especially for navigating complex parking maneuvers in cluttered and dynamic real-world environments. The inherent ability of these AI paradigms to learn from data and manage high-dimensional state-action spaces makes them increasingly suitable for the nuanced demands of modern APS.

At the same time, the performance and reliability of advanced AI methodologies are intrinsically contingent upon concurrent developments in sensor suite architectures, as the availability of high-resolution, multimodal data streams serves as a foundational enabler for robust perception, context-aware reasoning, and real-time decision-making in autonomous systems. Also, Table 6 shows the growing adoption of Solid-State LiDAR (SSL) for its improved reliability and cost-effectiveness [64], 4D imaging radar for its enhanced environmental understanding (including velocity data) [62], and specialized camera systems (thermal, event-based, multi-spectral) designed for robust perception in challenging conditions [64]. The rich, high-density data provided by these advanced sensors facilitates more detailed and reliable state representations, which are crucial for training effective DRL agents and other data-driven perception models. This co-evolution of sensor technology and AI algorithms is a critical enabler for progress in APS.

### 5.4. Identified Gaps and Future Research Pointers from Comparative Analysis

In fact, Table 6 provides impressive performance metrics and innovative solutions, a deeper examination suggests areas where current approaches may have limitations and where future research is needed. Notably, although many state-of-the-art AI-based planning systems report high success rates and notable efficiency improvements, primary research publications often place less emphasis on formal safety verification and comprehensive adherence to SOTIF (Safety of the Intended Functionality) standards. Then, it may indicate a common research trajectory where achieving peak functional performance with complex AI is an initial priority, with the development and integration of rigorous safety assurance methodologies for these newer, often black-box, techniques still an evolving frontier. This potential gap highlights the need for ongoing research into verifiable and interpretable AI for safety-critical applications, such as autonomous parking. Ensuring robust performance across a truly exhaustive range of Out-of-Distribution (OOD) scenarios and developing universally accepted, comprehensive validation frameworks for highly adaptive learning-based APS remains a significant challenge.

## 6. System Aspects and Emerging Challenges

Beyond the core algorithms for perception, planning, and control, the successful deployment of APSs involves addressing complex system-level integration, rigorous and comprehensive testing and validation methodologies, and tackling emerging challenges related to cybersecurity, user trust, and operational complexities in diverse real-world settings.

### 6.1. System Integration, Simulation, and Validation

Integrating the various software components (perception, planning, control, HMI) with the vehicle’s hardware platform (sensors, actuators, ECUs) is a complex engineering task. Research also explores hardware aspects, such as the utilization of powerful System-on-Chip (SoC) platforms that integrate Field-Programmable Gate Arrays (FPGAs) for parallel processing and ARM processor cores to handle demanding real-time computations [10]. Methodologies like Model-Based Systems Engineering (MBSE) and digital engineering workflows are being explored to manage the complexity of designing, developing, and validating these integrated autonomous systems systematically [4].

Simulation plays an indispensable role in the development lifecycle of APSs. Various simulation environments are employed, ranging from robotics simulators like Gazebo to specialized automotive simulators like CARLA and high-fidelity vehicle dynamics simulators like CarSim, often co-simulated with sensor simulators (e.g., for radar or LiDAR) and control design tools like MATLAB/Simulink [87,119]. Simulation enables rapid prototyping, cost-effective testing of algorithms under a wide range of reproducible conditions (including scenarios that cannot be safely tested in the real world), and systematic evaluation of system performance.

While simulation is crucial, validating APS performance in the real world is essential for ensuring safety and reliability [4]. A significant trend observed in recent literature is the increased emphasis on real-vehicle testing [12,13,14,80]. Researchers are deploying and evaluating their algorithms on actual vehicle platforms, often instrumented with research-grade sensors and drive-by-wire capabilities, in controlled test environments or real parking lots. Standardized test scenarios and performance evaluation frameworks are being developed to allow for more systematic and comparable assessment of different APS technologies [119].

The increasing emphasis on real-vehicle testing is crucial, yet the ‘reality gap’—the often significant difference between performance in simulation and performance in the physical world—persists and poses a significant challenge. Critically analyze why this gap is so challenging to close. Are current simulation tools [7,87], despite their increasing sophistication, capable of faithfully reproducing the full spectrum of sensor noise (e.g., thermal noise, interference, multipath reflections for radar), complex material reflectivity variations (e.g., how different paints, wet surfaces, or dirty vehicles affect LiDAR returns), intricate lighting interactions (e.g., dappled sunlight through trees creating complex patterns, reflections from wet pavement), and the nuanced, often unpredictable, and sometimes irrational behavior of other road users (pedestrians, cyclists, other drivers) encountered in real parking lots? If not, what are the limits of what can be reliably validated in simulation versus what must be validated extensively and expensively in the real world? And how can the efficiency and coverage of real-world testing be maximized to address the combinatorial explosion of potential scenarios without incurring prohibitive costs or timelines?

While efforts towards standardized test scenarios (e.g., predefined parking maneuvers, obstacle configurations) are noted and valuable for benchmarking [119], how comprehensive can these be in covering the combinatorial explosion of real-world variations in environmental conditions, parking lot layouts, vehicle types, and dynamic actor behaviors? Is there a risk of ‘teaching to the test,’ where systems are optimized to perform well on these specific standardized scenarios but lack generalized robustness when faced with novel situations not included in the test suite? Over-reliance on a limited set of standardized tests might create a false sense of security regarding a system’s overall real-world capabilities and safety.

### 6.2. Security Vulnerabilities in APS

As vehicles become more connected and automated, relying on complex software and communication networks, cybersecurity emerges as a critical concern [120]. APSs, especially those involving connectivity, such as AVP systems (which may communicate with infrastructure, remote operators, or user smartphones), are potential targets for malicious attacks. In-vehicle networks, such as the Controller Area Network (CAN) bus, are known to have vulnerabilities (e.g., lack of authentication or encryption) that could be exploited through attacks like message injection or denial-of-service, potentially compromising vehicle control by manipulating steering, braking, or acceleration [120]. Robust security measures, including Intrusion Detection Systems (IDSs)—potentially leveraging machine learning techniques to identify anomalous network traffic or sensor data—and secure software development practices are needed. Blockchain technology has also been proposed to enhance security and transparency in related areas, such as smart parking management systems that interact with APS, particularly for functions like automated fee calculation or access control [68].

So, in the case of fully autonomous valet parking (AVP) systems, which inherently depend on broader connectivity, including cloud services, parking infrastructure, remote human oversight, and user-facing mobile applications, the attack surface extends well beyond the confines of in-vehicle networks [120]. What are the unique, critical vulnerabilities introduced by these external communication links? Examples could include spoofing infrastructure signals (e.g., falsely indicating a parking spot is free or occupied), intercepting or replaying remote commands for vehicle movement, denial-of-service attacks on parking facility management systems or the AVP service itself (rendering vehicles unable to park or retrieve), or exploiting vulnerabilities in user authentication mechanisms to gain unauthorized access. What are the potential cascading failures if an AVP system’s security is compromised—could it lead to widespread vehicle immobilization within a garage, unauthorized vehicle movement causing damage or theft, large-scale data breaches of user information, or even the creation of “botnets” of compromised vehicles? Are current automotive cybersecurity paradigms, often focused on securing the vehicle itself, adequately addressing these distributed, networked threats that span multiple entities and communication channels? The security focus must extend beyond the individual vehicle to encompass the entire AVP ecosystem, including backend servers, communication protocols, and infrastructure components.

### 6.3. User Interaction, Acceptance, and Trust

Technological capability alone does not guarantee the success of APS; user acceptance and trust are crucial factors influencing adoption and effective use [5,117,121]. Public perception can be negatively impacted by reports of AV accidents, even if unrelated to parking, and concerns about safety, reliability, and loss of control remain significant barriers [5]. Factors influencing acceptance include perceived ease of use, usefulness, safety, risk, and the perceived competence of the system. A specific concern identified is Human-Manipulated Risk Perception (HMRP)—the perceived risk associated with scenarios requiring human intervention or oversight in partially automated systems, which can negatively impact user attitudes and their sense of control [5].

Building user trust requires the careful design of the Human–Machine Interface (HMI) and the leveraging of Explainable AI (XAI) to make the system’s behavior transparent and understandable [117,118]. Recent research has investigated scenario-based explanation frameworks for AVP systems. Studies show that providing clear, timely, and relevant explanations for the AV’s decisions and actions (e.g., “waiting for pedestrian to cross,” “adjusting path for narrow space”) via the HMI can significantly improve driver trust, enhance the overall user experience (UX), reduce the mental workload associated with monitoring the system, and even improve objective performance by enabling users to anticipate system actions [117,118]. Importantly, the effectiveness of explanations can vary depending on the user’s familiarity with the technology and their cognitive style. Customizing explanations—providing more detailed guidance and transparency for new or anxious users, while focusing on efficiency and critical safety information for frequent, confident users—has been found to be significantly more effective and satisfying than generic, one-size-fits-all explanations [117,118].

While XAI aims to enhance transparency and thereby foster trust, what are the limitations of current explanation methods, especially when attempting to explain the complex, often emergent behavior of deep learning systems or the nuanced decisions resulting from multi-objective optimizers in path planning? Can an explanation that is itself an approximation, a simplification, or a post hoc rationalization of an opaque process truly foster deep, resilient trust, particularly if the system still makes occasional, inexplicable errors that contradict the provided explanations? Superficial explanations might even erode trust if they are perceived as unhelpful or misleading. Beyond HMI design and XAI, what are the deeper socio-technical factors (e.g., perceived loss of control and agency, ethical concerns about algorithmic decision-making in potential conflict scenarios even in low-speed parking, negative media narratives shaping public opinion, lack of clear liability frameworks in case of malfunction, or anxieties about data privacy) that create persistent barriers to widespread acceptance [5]? How can these be addressed systemically—through public education, transparent development practices, robust regulatory frameworks, and ethical guidelines—rather than just at the interface level? True trust requires more than just a well-designed dashboard explanation; it involves a broader societal adaptation, clear accountability, and consistent, reliable system performance over extended periods.

### 6.4. Operational Challenges

Several operational challenges continue to impede the development and deployment of fully robust and universally applicable APSs. Operating reliably in diverse and unstructured environments, including those with poor or non-existent markings, unusual geometries (e.g., angled parking on steep slopes), or during adverse weather conditions that degrade sensor performance, remains profoundly difficult. A significant contributing factor, as previously highlighted, is the lack of large-scale, diverse, and publicly available datasets for training and benchmarking perception and planning algorithms [7,72]. This is particularly acute for end-to-end learning approaches, which are exceptionally data-hungry, and for rigorous robustness testing under a wide array of adverse conditions. Additionally, coordinating multiple autonomous vehicles within shared parking facilities, such as in multi-UGV (Unmanned Ground Vehicle) or AVP scenarios in large garages, presents significant challenges in path planning, real-time conflict resolution, efficient resource allocation (e.g., assigning parking spots or charging stations), and communication protocols [79].

## 7. Future Directions

Based on this critical review, future research should intensify its focus on the following key directions:

- End-to-End Learning with Verifiability and Safety Guarantees. A key emerging research direction is the development of end-to-end learning approaches that map sensor inputs directly to control actions. While still a nascent area for production systems, this paradigm holds significant potential for simplifying the traditional, modular APS pipeline and discovering novel, holistic solutions [7,72]. However, this approach intensifies challenges related to data dependency, interpretability, and safety verification. Future work must integrate mechanisms for robust interpretability, comprehensive uncertainty quantification, and, where possible, formal verification or runtime monitoring with safety fallbacks into these architectures.
- Robust Multi-Modal Perception and Advanced Fusion. Research into sensor fusion techniques that can gracefully handle sensor degradation or complete failure of one or more modalities, resolve conflicting information with high reliability using uncertainty-aware methods, and adapt dynamically to extreme environmental conditions is crucial [6,7,8,9,10,17,62,64,65]. This includes developing better domain adaptation methods and creating self-assessment capabilities within perception systems to flag low-confidence situations.
- Safety Verification and Validation (V&V) for AI-based Systems. Developing more rigorous, scalable, and widely accepted V&V methodologies specifically tailored for AI-driven systems is paramount. This includes advancing formal methods applicable to neural networks, investing in large-scale realistic simulation platforms with a strong focus on automated edge case generation, and establishing standardized safety metrics and benchmarks aligned with automotive safety standards like ISO 26,262 and SOTIF (ISO 21448) [4,119,122,123,124].
- Human-Centric APS Design and Trust Calibration. Deeper investigation into XAI techniques that provide causal, contrastive, and actionable explanations is needed [117,118]. Research should focus on adaptive HMIs that manage user expectations and reduce cognitive load. Longitudinal studies on trust dynamics—how trust is built, lost, and potentially repaired over time—are needed to ensure users neither dangerously over-trust an imperfect system nor under-utilize its capabilities [5].
- Cooperative and Multi-Agent Systems with Scalable Coordination. As vehicle connectivity (Vehicle-to-Everything (V2X)) increases, research into decentralized, robust, and scalable algorithms for coordinating multiple autonomous vehicles in shared parking environments (e.g., efficient allocation of spots, collision-free maneuvering, negotiation for shared resources) will become increasingly important [79,120,125].
- This includes not only vehicle-to-vehicle (V2V) coordination but also vehicle-to-infrastructure (V2I) cooperation, which presents significant opportunities. For instance, future work could explore smart parking lots that communicate directly with vehicles to guide them to available spots, or cloud services that offload some of the complex computational logic for path planning, reducing the burden on the vehicle’s onboard systems.
- Proactive Cybersecurity for Connected APS Architectures. Dedicated research is urgently needed to identify specific vulnerabilities in distributed APS architectures—spanning sensors, ECUs, V2X links, and backend cloud infrastructure—and to develop tailored, adaptive, and resilient intrusion detection and prevention mechanisms [68,120,125].
- Addressing the Data Bottleneck Systematically and Collaboratively. Concerted efforts towards creating large-scale, high-quality, diverse, and well-annotated public datasets are essential [7,65,71,72]. This may involve exploring novel data collection strategies, advancing synthetic data generation techniques, and investigating federated learning approaches.

Addressing these research directions with a critical, holistic, and collaborative perspective will be key to overcoming the remaining hurdles and realizing the full societal and economic benefits of Autonomous Parking Systems in future mobility solutions, ultimately leading to safer, more efficient, and less stressful parking experiences

## 8. Conclusions

This review has charted the significant evolution of Autonomous Parking Systems (APS), highlighting a clear trend towards AI-centric solutions. Deep learning now forms the foundation of perception, while hybrid algorithms that pair search-based methods with optimization techniques dominate path planning. In vehicle control, there is a notable shift toward learning-based strategies like reinforcement learning to enhance adaptability. The field is also maturing beyond pure algorithms, with an increasing emphasis on real-world vehicle testing and a growing recognition of the criticality of user trust, as evidenced by research into explainable AI and human–machine interfaces.

Despite this progress, formidable challenges persist. The primary obstacle remains achieving robust system performance across the full, unpredictable spectrum of real-world conditions, from adverse weather to poorly maintained infrastructure and erratic human behavior. Guaranteeing the safety and security of opaque AI components, closing the persistent gap between simulation and reality, and overcoming public skepticism are critical hurdles. The scarcity of comprehensive public datasets continues to be a fundamental bottleneck, hindering the development of truly generalizable AI. Ultimately, while the technological building blocks of APSs have advanced considerably, the path to deploying fully reliable, safe, and trusted systems requires a continued, concerted effort to solve these deep-seated challenges.

## Figures and Tables

**Table 1:** Critical Analysis of Sensor Technologies in Autonomous Parking Systems (APS) ^1^.

Sensor Technology	Key Reported Strengths	Inherent Weaknesses/Practical Limitations	Specific Fusion Challenges	Cost vs. Performance Considerations
Standard Cameras (RGB, Fisheye, Rectilinear)	- Rich semantic info (color, texture) for classification, recognition, identification [18]. - Fisheye: Wide FoV for near-vehicle sensing [19]. - Cost-effective; foundational ADAS/APS component [18]. - Note: Performance limits are a system challenge. - Advanced AI/DL support; mature algorithms [18]. - Passive sensing; avoids interference [18].	- Illumination dependent: Degrades in low light, glare, shadows [18]. Critical for APSs in garages/night. - Susceptible to adverse weather (rain, fog, snow) [20]. - Limited direct depth (monocular); stereo adds cost/complexity [18]. - Lens occlusion/soiling: Critical reliability issue [18,21]. - Fisheye distortion: Requires correction, adds overhead [22].	- Calibration (intrinsic/extrinsic) with 3D sensors critical [20]. - Temporal synchronization with other sensors essential [20]. - Data association: Matching features/objects across sensors. - Resolving conflicting data from different sensor types. - Handling sensor degradation (soiling, occlusion) and adapting and fusion [21,23].	- Low unit cost: Enables multi-camera 360° APS [18]. - High processing cost: Rich data needs powerful ECUs (Electronic Control Units)/SoCs [22]. - Data deluge can be a bottleneck. - Central to basic APSs (with ultrasonics); limited in diverse conditions [22]. - Insufficient alone for advanced APS safety/ODDs (Operational Design Domains); needs fusion [21,22].
Ultrasonic Sensors	- Extremely low cost: Ubiquitous for basic parking aid [24]. - Reliable short-range detection (<5–10 m): Crucial for final maneuvers, low obstacles [18]. - Acts as “last-centimeter” guardian. - Independent of lighting conditions [18]. - Detects various material types (large, hard surfaces) [18].	- Limited range (very short distances) [18]. - Poor object classification/identification; only distance [18]. - Sparse data, hard to fuse intelligently. - Narrow FoV per sensor; multiple units needed, may leave gaps [25]. - Susceptible to environment (wind, temp, heavy rain/snow) [18]. - Struggles with soft, curved, thin, small, sound-absorbing objects [18]. - Contamination/damage: Susceptible to dirt, ice; silent failures possible [26].	- Complementary fusion only: Provides short-range safety bubble [19]. - Data association: Linking sparse pings to rich camera/LiDAR data. - Handling false positives/negatives: Robust filtering needed. - Sensor degradation detection (obstruction/damage) important [26,27]. - Self-check or cross-validation needed.	- Extremely low cost: Main driver for adoption [24]. - Essential for low-speed maneuver safety [28]. - Not standalone for advanced APS; part of larger suite [19].
Solid-State LiDAR (SSL) (e.g., MEMS, OPA, Flash)	- Improved reliability/durability: No/fewer moving parts [29]. - Compact size and lower weight: Easier vehicle integration [29]. - Potential for lower cost (mass production) [30]. - “Democratization” of LiDAR is key. - High data rate/resolution: Detailed 3D mapping/detection [29]. - Fast scanning (some types); Flash LiDAR illuminates entire scene [30]. - Good performance in various lighting (active sensor) [18].	- Limited FoV per unit: Multiple units often needed for 360°, offsetting cost benefits [31]. - Shorter range (historically/certain types), though improving [29]. - Adverse weather performance degraded (rain, snow, fog) [18]. - Thermal management challenges: Heat build-up affects performance/durability [31]. - Near-field detection issues/blind spots (sub-meter to few meters) for some types [32]. - Irregular scan patterns (some MEMS): Can complicate processing [33].	- Calibration of multiple SSL units: Crucial and complex [20]. - Data synchronization: Multiple SSLs and other sensors. - Point cloud registration/stitching: Merging data from multiple SSLs. - High data bandwidth and processing demands [20]. - Handling sensor degradation (soiling, thermal effects).	- Current cost still a factor, especially for multiple units [29]. - Trade-off: FoV/Range vs. Cost (lower-cost SSLs may be limited). - Performance benefits for APS: High resolution/accuracy for small obstacles, mapping, localization [30]. - Mass-market viability target: Achieving performance at suitable price [34].
4D Imaging Radar	- Adverse weather robustness: Excellent in rain, fog, snow [18]. - Crucial baseline sensor for APS continuity. - Direct velocity measurement (Doppler) [18]. - Elevation information (4th D): Better 3D object detection/classification [35]. - Long detection range [36]. - Can “see through” some non-metallic obstructions [35]. - Improved angular resolution (vs. traditional radar) [36].	- Lower resolution than LiDAR/Cameras: Difficult for detailed classification/boundaries [18]. - Point cloud sparsity (vs. LiDAR) [37]. - Noisy data/clutter/multipath: Especially in dense metallic environments (garages) [18]. - Limited material differentiation (no color/texture) [18]. - Challenges with stationary objects (differentiation) [35]. - Frequency regulation hurdles for higher resolution [35]. - Short-range blind spots (ground reflection/DC noise) [35].	- Fusing sparse radar with dense camera/LiDAR: Sophisticated techniques needed [38]. - Calibration and Synchronization: Precise spatio-temporal alignment crucial [20]. - Resolving conflicting detections. - Computational load for raw 4D radar tensor processing (e.g., RadarOcc [37]). - Handling sensor degradation.	- Higher cost for high performance (vs. traditional radar/cameras); projected cheaper than LiDAR [35]. - Production costs a barrier [39]. - Single-chip solutions aim to reduce cost for mass-market [40]. - Value for APS: All-weather, velocity, elevation data for reliability [35]. - Trade-off: Resolution vs. Cost (higher-res is more expensive) [35].
Event-Based Cameras (DVS, DAVIS)	- High temporal resolution (µs-scale): Captures fast dynamics (e.g., sudden pedestrian) [38]. - Low latency (µs to sub-ms): Critical for reactive APSs [38]. - High Dynamic Range (HDR >120 dB): Adapts to extreme lighting (garage entry/exit) [38]. - Reduced motion blur [38]. - Potential low power consumption (data only on change) [38]. - Data sparsity: Efficiently represents dynamic scenes [41].	- No/Limited static scene info: Major limitation for APSs (stationary obstacles/lines) [38]. - Positioned as “dynamic specialists.” - Grayscale info: Typically event polarity, not absolute intensity/color (DAVIS adds frames) [38]. - Noisy events/data interpretation: Requires specialized algorithms [38]. - Lack of inherent semantic info [41]. - High data volume (highly dynamic scenes): Can negate low-bandwidth advantage [41]. - The “data paradox.” - Maturity and Cost and: Emerging tech, cost may be higher, algorithms less mature [38].	- Fusing asynchronous data with synchronous frame-based data: Significant challenge [19]. - Requires new algorithmic paradigms (change-driven). - Event representation for fusion: Converting sparse events can lose info/add cost. - Calibration: Precise spatio-temporal needed. - Complementary fusion for static scenes: Heavily rely on frame-based cameras/LiDAR [42].	- Higher unit cost (currently) vs. standard CMOS cameras [38]. - Potential system-level savings (low power/data in some scenarios)—depends on processing. - Niche performance benefits: Unmatched HDR/high-speed. Useful for sudden intrusions/extreme lighting in APS. - Not a standalone APS solution: Due to static scene issues; more for specialized dynamic threat detection.
Thermal Cameras (LWIR)	- Low/No visible light detection: Operates in complete darkness (e.g., underground garages) [18]. - “Lifesaver” for detecting pedestrians/animals when visible cameras fail. - Robustness to visual obscurants (smoke, haze, light fog) [43]. - Good for detecting animate objects (pedestrians/animals via heat) [43]. - Reduced glare issues (sunlight/headlights) [28]. - High contrast imaging (for warm objects) [43].	- Lower resolution vs. visible cameras [44]. - Lack of color and fine and texture: Grayscale (temperature-based); no detailed visual ID [43]. - “What, not who” limitation. - Cannot see through glass/water [43]. - Temperature-dependent contrast: Image quality affected by ambient/object temp difference [43]. - Difficulty with isothermal scenes/cold obstacles [43]. - Higher cost than standard cameras [43].	- Fusing low-res thermal with high-res visible: Requires careful registration/scaling. - Different data modalities: Fusing heat-based with reflection-based (visible, LiDAR) images. - Calibration and Synchronization and: Precise spatio-temporal essential [45]. - Complementary role: Often supplements visible cameras (low-light pedestrian detection) [45]. - Fusion logic needs to weigh inputs based on conditions.	- Higher cost component: Increases sensor suite cost [46]. - Cost–benefit often positions for premium APS/specific ODDs. - Significant performance boost in specific APS scenarios (dark lots, underground, missed pedestrians/animals) [46]. - Niche capability vs. Cost trade-off: Depends on target ODD/safety requirements. - Deep Learning for Thermal (e.g., YOLO-Thermal [44]) aims to improve performance.
Multispectral Cameras (RGB + NIR, Red Edge, etc.)	- Improved object/material discrimination: Captures multiple spectral bands (visible, NIR, SWIR) for detailed material analysis [47]. - Potential “surface condition specialist” (black ice, oil slicks with SWIR). - Enhanced performance in specific conditions (e.g., NIR for light haze/fog penetration) [47]. - Simultaneous multiband imaging (snapshot cameras) for dynamic scenes [48].	- Higher cost and complexity and vs. standard RGB cameras [47]. - Data volume and processing and: Multiple bands increase data/computational load [48]. - Data richness can be “curse of dimensionality.” - Limited bands vs. hyperspectral: Less finesse in spectral analysis [47]. - Application-specific band selection: General APSs might not leverage all specialized bands. - Illumination dependent: Still reliant on external/active illumination in specific bands.	- Registration of multiple bands: Ensuring perfect alignment crucial (snapshot cameras mitigate) [48]. - Fusion with other sensor modalities: Integrating multiband with LiDAR, radar, thermal. - Feature extraction and selection: Identifying relevant spectral features for APSs (e.g., wet vs. dry pavement). - Real-time processing: Can be computationally demanding [49].	- High cost for automotive integration: Likely prohibitive for mass APS [47]. - Niche APS benefits: E.g., road surface condition detection. - Justifying performance gain vs. Cost: Incremental gain must be significant. - Visible–Infrared focus: Current research often on visible–IR fusion for object detection [49]. - Could be cost–performance step if key bands identified for critical APS issues.

^1^ This table presents a critical analysis of sensor technologies in Autonomous Parking Systems (APSs), synthesized from the information provided in the correspondingly cited references listed within the table (in the ‘Critical References’ column). For definitions of abbreviations used, please refer to the ‘Abbreviations’ section at the end of the manuscript. Italicized text or sub-bullets within table cells provide supplementary commentary or specific details.

**Table 2:** Critical Analysis of Sensor Fusion Approaches in Autonomous Parking Systems (APS) ^2^.

Sensor Fusion Approach	Key Reported Strengths	Inherent Weaknesses/Practical Limitations	Specific Fusion Challenges	Cost vs. Performance Considerations
Tightly-Coupled LiDAR-IMU Fusion (e.g., LIO-SAM, FAST-LIO, GF-LIO)	- Improved SLAM (Simultaneous Localization and Mapping)/Odometry: Higher accuracy/robustness in state estimation/mapping, especially GPS-denied (garages) [50]. - De facto standard for robust localization in critical APS ODDs. - Motion distortion correction: IMU de-skews LiDAR clouds [50]. - Enhanced state estimation (degenerate scenarios): IMU aids when LiDAR data sparse [50]. - Improved attitude estimation (roll, pitch, yaw) [51]. - Real-time capability with modern algorithms [50].	- Computational complexity: Joint optimization is intensive [50]. - Balancing accuracy vs. real-time on embedded hardware. - Sensitivity to initialization and calibration: Small errors amplified [50]. - IMU noise and bias drift: Needs accurate online estimation [52]. - Dependence on accurate sensor models [53].	- Precise spatio-temporal calibration (LiDAR-IMU) [20]. - Complexity of IMU preintegration [50]. - Factor graph optimization management (size/complexity) [50]. - Loop closure integration with LIO factors [50]. - Handling reflective/symmetric structures (LiDAR-specific garage challenges) [51,54].	- Adds IMU cost (higher-grade IMUs improve performance but cost more). - Significant performance gain for APS localization/mapping in GPS-denied areas [54]. - Enables higher automation levels (prerequisite for precise, continuous localization). - Computational cost implication: May need more powerful ECUs.
BEV (Bird’s Eye View) Fusion (Cameras, LiDAR, Radar)	- Unified spatial representation: Common top-down view for fusing heterogeneous data [55]. - BEV as “lingua franca” for sensor modalities. - Facilitates multimodal fusion: Combines features from different perspectives/structures [55]. - Improved situational awareness: 360° view for navigation/decision-making [56]. - Potential for dense fusion: Preserves more contextual info [57]. - Directly applicable to planning: BEV maps usable by motion planners.	- Information loss in view transformation: Projecting to BEV can cause distortion, ambiguity (distant/occluded objects) [7]. - Lack of depth-aware vision-to-BEV can cause gaps. - Standard 2D BEV loses height info; drives research to 3D/pseudo-3D BEV. - Handling occlusions: Difficult, especially with camera-only systems [55]. - Computational cost: Generating/processing BEV from multiple high-res sensors can be intensive [56]. - Dependence on accurate calibration and synchronization [20]. - Fixed grid resolution: Limits accuracy vs. computational load.	- Camera-to-BEV transformation: Accurate projection, often needs depth estimation or learned transformers (e.g., LSS) [7]. - LiDAR/Radar-to-BEV representation: Efficiently projecting sparse/dense data. - Cross-modal feature alignment and fusion: Camera appearance, LiDAR geometry, radar velocity [55]. - Temporal fusion in BEV: Incorporating history to improve consistency/handle occlusions [7]. - Synchronization delays for multi-camera BEV (RT-BEV aims to mitigate) [56]. - Handling sensor degradation: Affects fused BEV quality.	- Sensor suite cost (multi-camera, LiDAR, radar). - Vision-centric BEV aims for lower cost but faces performance limits [56]. - Computational hardware: Requires powerful ECUs/SoCs with GPUs [56]. - Performance gains for APS: Holistic understanding for complex maneuvers, spot finding, clutter navigation [19]. - Scalability: Sensor number/resolution impacts performance/cost.
Probabilistic Fusion Frameworks (e.g., Kalman Filters, Particle Filters, Bayesian Networks, Occupancy Grids)	- Uncertainty management: Explicitly model/manage sensor noise, environmental variability, model inaccuracies [20]. - Crucial for safety-critical APS decisions. - Conflict resolution: Principled ways to fuse conflicting info by weighting data based on reliability/uncertainty [20]. - Improved SNR and fault tolerance (Raw Data Fusion) [58]. - State estimation and tracking: Kalman/Particle filters widely used [20]. - Semantic mapping: Probabilistic generation of semantic maps (road, curb) with confidence levels [59]. - Occupancy Grids: Represent free space/obstacles probabilistically for APS path planning [58].	- Model dependence: Performance relies on accuracy of system/sensor models [20]. - Computational complexity: Some methods (Particle Filters for high-dim, full Bayesian) very expensive for real-time APS [20]. - Assumptions (e.g., Gaussian noise for LKF/EKF): May not hold in real-world APSs [60]. - Data association challenges: Associating measurements to tracks/map features in clutter. - Scalability: Maintaining real-time performance with increasing objects/map size.	- Data alignment and synchronization: Critical prerequisite [20]. - Handling heterogeneous data: Integrating diverse sensor types (point clouds, images, radar) with different noise/resolutions [20]. - Dynamic noise covariance estimation: Adaptive estimation important but challenging [28]. - Non-linearities and Non-Gaussianities: Standard KFs struggle; need EKF, UKF, Particle Filters (with own trade-offs) [61]. - Computational latency for high-bandwidth data.	- Trade-off: Robustness vs. Computational Cost (sophisticated models cost more). - Enabling safer decisions: Quantifying uncertainty allows more informed APS actions. - Development cost: Implementing/validating complex probabilistic fusion requires expertise/testing. - Use of lower-cost sensors (with Raw Data Fusion): May enable by improving SNR/overcoming individual failures.

^2^ This table presents a critical analysis of sensor fusion approaches in Autonomous Parking Systems (APSs), synthesized from the information provided in the correspondingly cited references listed within the table (in the ‘Critical References’ column). For definitions of abbreviations used, please refer to the ‘Abbreviations’ section at the end of the manuscript. Italicized text or sub-bullets within table cells provide supplementary commentary or specific details.

**Table 3:** Comparison of recent DL-based parking slot detection Methods ^3^.

Method Category	Specific Method/Key Innovation	Dataset(s) Used	Key Performance Metrics Reported	Robustness Aspects Addressed/Target Platform	Reference(s)
Object Detection (YOLO)	Improved YOLOv5-OBB (Oriented BBox, Backbone opt., CA mechanism)	Homemade	mAP +8.4%, FPS +2.87, Size −1 M	Lighting variations; low-compute embedded	[3]
Object Detection (YOLO)	YOLOv5 (Fine-tuned)	PKLot, Custom	Valid. Acc: 92.9%	Real time (PARKTag system)	[68]
Hybrid (DL + CV)	Two-Stage: YoloV11 (Key points) + CV (Special kernel for rotation)	ps2.0 (Public)	Acc: 98.24%, Inf. Time: 12.3 ms (Desktop), 16.8 ms (Laptop)	Speed; varied cond. (ps2.0)	[11]
Segmentation	Mask R-CNN	Tongji Parking-slot DS	Precision: 94.5%, Recall: 94.5%	Lighting variability; occlusions	[69]
Segmentation	Novel Convolutions (Directional, Large Field) for low-level features	Public Remote Sensing	Improved perf. vs. baseline	Potential for parking	[73]
End-to-End Learning	LSS-based Transformer/BEVFusion (Camera -> Control)	CARLA (Simulated)	Success: 85.16%, Pos Err: 0.24 m, Orient Err: 0.34 deg	End-to-end pipeline, dataset creation	[7]

^3^ This table provides a comparison of recent deep learning (DL)-based parking slot detection methods, synthesized from the information provided in the correspondingly cited references listed within the table (in the ‘Reference(s)’ column). For definitions of abbreviations used, please refer to the ‘Abbreviations’ section at the end of the manuscript. Italicized text or sub-bullets within table cells, if any, provide supplementary commentary or specific details.

**Table 4:** Overview of recent APS path planning algorithms ^4^.

Category	Specific Method/Combination	Key Features	Constraint Handling	Smoothing Method	Validation	Reference(s)
Search + Opt.	Hybrid A* + NMPC	- Hierarchical - NMPC optimizes coarse path	- Narrow spaces - Kinematics	Implicit (NMPC)	Sim, Real	[13]
Search + Opt.	Hybrid A* + QP Smoothing + S-Curve Speed Planning	- Adaptive search - Improved heuristic - QP smoothing - S-curve speed	- Constrained env. - Kinematics	- QP - S-Curve	Sim, Real	[14]
Search + Opt.	Graph Search (Hybrid A*) + Numerical Opt. + I-STC	- Hierarchical - Warm start - I-STC simplifies collision constraints	- Parallel parking - Narrow spaces	Numerical Opt.	Sim	[81]
Search + Opt.	Hybrid A* + GA (Genetic Algorithm) Opt. + Geometric Curves (Bézier and Clothoid)	- Hierarchical - GA local opt. - Curve smoothing	- Tight spaces - AVP	- Bézier - Clothoid	Sim	[76]
Search + APF	RRT* + Improved APF + Variable Probability Strategy	- Guided sampling - APF avoids local minima - Adaptive sampling	Obstacle avoidance	Enhanced APF	Sim	[77]
Optimal Control	Indirect OCP (Minimum Time) + pNN Controller	- Efficient OCP solver (PINS) - Smooth 3D penalty functions - Complex maneuvers	- Narrow spaces - Unstructured - Kinematics	Implicit (OCP)	Sim, Real	[12]
Prediction + Opt.	Prediction + APF + Bézier Curve + SQP Optimization	- Integrates prediction - APF target selection - SQP optimizes Bézier CPs	- Dynamic obstacles - Collision avoidance	Bézier Curve + SQP	Sim	[16]
Reinforcement Learning	Soft Actor-Critic (SAC) + Segmented Training Framework	- Optimizes safety, comfort, efficiency, accuracy - Handles neighbor deviation	- Non-ideal scenarios - Kinematics	Implicit (Learned Policy)	Sim	[15]
Reinforcement Learning	Hybrid RL (Rule-based RS + Learning-based Planner)	- Combines rule-based feasibility with learned adjustments	Complex environments	Implicit (Learned Policy)	Sim, Real	[82]
Multi-Agent	Improved Conflict-Based Search (ICBS) + Swarm Opt. (IACA-IA) + Adaptive A*	- Multi-UGV coordination - Conflict resolution - Slot allocation	- High-density - Multi-vehicle	Adaptive A*	Sim	[79]

^4^ This table provides an overview of recent Autonomous Parking Systems (APSs) path planning algorithms, synthesized from the information provided in the correspondingly cited references listed within the table (in the ‘Reference(s)’ column). For definitions of abbreviations used, please refer to the ‘Abbreviations’ section at the end of the manuscript. Italicized text or sub-bullets within table cells, if any, provide supplementary commentary or specific details.

**Table 5:** Critical evaluation of vehicle control strategies in Autonomous Parking Systems (APSs) ^5^.

Control Strategy	Operating Principles	Reported Advantages for APS	Verification/Safety and Robustness Challenges	Handling Precision vs. Comfort/Efficiency Trade-Offs
Model Predictive Control (MPC)—Standard	- Explicit vehicle model (e.g., bicycle [88]) predicts future states (Np) [89]. - Optimizes control sequence (Nc) by minimizing cost function (tracking error, effort, constraints) [88]. - Applies first control input; repeats (receding horizon) [90]. - Explicitly handles system constraints (actuator/state limits, obstacle avoidance) [89,90].	- High precision/accuracy for tight spaces [90]. - Optimal performance and constraint handling (vehicle limits, obstacles) [89]. - Improved comfort (smoother motion vs. simpler controllers) [91]. - Enhanced robustness (vs. some traditional methods) [91]. - Predictive capability: Proactive control [89]. - Solves constrained motion problems optimally [46].	- Model fidelity: Performance/safety depend on model accuracy; mismatches problematic [89]. - Obtaining perfect models for all conditions is challenging. - Computational complexity: Real-time optimization can be demanding [89]. - Recursive feasibility: Ensuring a solution exists at every step is a major challenge [92]. - Tuning complexity: Many parameters (weights, horizons) need careful tuning [46]. - Uncertainty handling: Standard MPC assumes deterministic models; robust variants increase complexity/conservatism [92].	- Adjusting weights in cost function (Q,R,P) [88]. - Precision: ↑ weight on tracking error. - Comfort: Penalize aggressive inputs (steering rate, acceleration). - Efficiency: Penalize path length/time. - Hard constraints for precision; soft for comfort/feasibility. - Prediction/control horizons ($N_{p},N_{c}$) influence behavior.
Learning-Based MPC (e.g., residual models, Koopman, GP, NN-adaptive)	- Integrates ML to improve prediction model or compensate for deficiencies [89]. - Residual Model Learning: Learns unknown dynamics (GP, NN) to augment nominal model [89]. - Koopman Operator Theory: Lifts nonlinear dynamics to linear space for prediction [93]. - NN-Adaptive LPV-MPC: NN adapts LPV-MPC parameters online (e.g., tire stiffness) [44].	- Enhanced model accuracy and control performance [89]. - Adaptability to varying conditions (road friction, tires) [46]. - Reduced dimensionality for learning (residual models) [89].	- Safety of learned components (GP, NN): Formal verification difficult [93]. - Data requirements and generalization: Poor OOD performance risks safety [89]. - Verification of hybrid system (MPC + learned parts). - Computational overhead of online learning/adaptation. - Defining valid feature space for learned component to avoid unsafe extrapolation [89].	- MPC cost function still key for balance. - Learned component might implicitly balance if trained on such data. - Economic MPC (EMPC): Cost function directly optimizes efficiency (e.g., energy [94]) alongside performance. - Learning helps achieve trade-offs if model is more accurate.
Reinforcement Learning (RL)—General	- Agent learns actions (steering, accel.) by interacting with environment (parking scenario) [95]. - Learns policy (state -> action) to maximize cumulative reward [95]. - Trial-and-error learning, often in simulation [95]. - Model-free (direct policy) or model-based (learns model) [49]. - End-to-end RL: Sensor inputs -> control commands [96].	- Adaptability to complex/dynamic environments [95,97]. - Learning complex maneuvers for tight spaces [98]. - Optimization through interaction; potential for novel solutions [95]. - Reduced reliance on hand-engineered rules [99].	- ‘Black box’ nature: Formal safety verification very difficult [95]. - Safety during learning (exploration): Mostly simulated, leading to sim-to-real gap [95]. - Sim-to-real transfer gap: Policies often perform poorly in real world [82]. - Bridging gap (domain randomization, OGM (Occupancy Grid Map) [82]) is critical. - Robustness to OOD states: Catastrophic failure possible [99]. - Reward hacking and specification: Defining correct reward function is hard [99]. - Stability and convergence issues [96]. - Data bias and sufficiency [95]. - Computational demands for training DRL [95].	- Primarily via Reward Function Design [99]: - Precision: Reward proximity/alignment; penalize collisions/deviations. - Comfort: Penalize jerk, high accel/decel, large steering rates. - Efficiency: Reward speed, short paths, low energy; penalize maneuvers. - Multi-Objective RL (MORL): Explicitly multiple rewards, balancing complex. - Constraint-driven safety RL (e.g., CMDP): Safety as hard constraint [99]. - Hierarchical RL: Different rewards per level (strategic vs. maneuver) [100]. - Reward changes can lead to unpredictable policy changes.
Reinforcement Learning (RL)—Soft Actor-Critic (SAC)	- Off-policy, model-free, actor-critic DRL for continuous actions [99]. - Actor (policy) decides actions; Critic (value/Q-net) evaluates [99,101]. - Entropy regularization: Maximizes reward + policy entropy (encourages exploration) [101]. - Off-policy learning from replay buffer (sample efficiency) [99,101].	- High parking success rates (simulated) [97]. - Reduced maneuver times (vs. traditional/other DRL) [101]. - Robust handling of dynamic obstacles [101]. - Fine-grained vehicle control (continuous actions) [101]. - Improved sample efficiency (with HER for sparse rewards) [97].	- Same general RL challenges: Black box, verification, safety, sim-to-real, OOD, reward, stability, data, computation [95]. - SAC properties may mitigate some training issues (sample efficiency, exploration) but fundamental safety challenges persist.	- Similar to general RL (reward function design). - Entropy regularization might naturally lead to smoother policies (comfort) [101]. - Explicit control over trade-offs still heavily relies on reward component formulation.
Neural Network (NN) Based Controllers—Direct NN Controllers (End-to-End)	- NN (often deep) maps sensor inputs (LiDAR [102], camera, sonar [103]) or states to control commands [102]. - Learns mapping from data (imitation learning [102]) or RL. - Internal layers learn hierarchical features. - NNs with ReLU: Complex piecewise linear functions [102]. - Sigmoid/tanh: Smooth non-linearities [104].	- Learning complex kinematics/dynamics from data [103]. - Automatic generation of control commands [103]. - Adaptability to different situations (if trained on diverse data) [103]. - Reduced parking time; system-level, connected CAVs (Connected Automated Vehicles ) [105]. - Handling high-dimensional inputs (e.g., LiDAR images) [102].	- ‘Black box’ and lack of interpretability: Hard to debug, provide safety guarantees [102]. - Formal verification difficulties: Computationally challenging for deep NNs [102]. - Techniques exist (hybrid systems, SMC, reachability) but have limits. - System-level safety (closed-loop) very complex. - Robustness to OOD states/adversarial attacks: NNs can be brittle [106]. - Robust OOD detection essential [107]. - Data dependency and generalization issues [106]. - Sim-to-real gap. - Lack of precise mathematical specifications for “correct” behavior [102].	- Implicitly learned from training data (imitation or RL reward). - If trained on human data, mimics that balance. - Hard to predictably adjust behavior post-deployment without retraining. - Neural abstractions trade abstraction precision vs. verification time [108].
Neural Network (NN) Based Controllers—Jordan Neural Networks	- Recurrent NN: Output fed back as input via “context units” [103]. - Inputs: Sensor readings, odometer, current maneuver state. - Outputs: Control commands (speed, steering), next maneuver state [103]. - Learns from examples of successful maneuvers (supervised learning, e.g., RPROP) [103].	- Automatic knowledge acquisition from examples [103]. - Adaptability and robustness: Potential generalization to new situations (vs. rigid rules) [103]. - Simplified development: Focus on collecting examples [103].	- Similar to direct NN: Black box, verification, data dependency, generalization, sim-to-real. - Recurrent structure adds complexity to formal analysis. - Ensuring stability/convergence of recurrent dynamics can be challenging.	- Trade-offs primarily learned implicitly from training examples. - Quality of demonstrated maneuvers dictates learned policy balance. - Explicit tuning post-training difficult without new data.
Hybrid RL Approaches—RL + Rule-Based Planners (e.g., RL with Reeds-Shepp (RS) curves, A*)	- Combines stability/guarantees of traditional planners (RS, A*) with RL’s adaptability [82]. - Rule-based planner: Initial reference/candidate paths [109]. - RL agent: Refines trajectory, selects candidates, or makes high-level decisions [82]. - E.g., RS path + RL speed/steering adjustments from LiDAR OGM [82].	- Improved generalizability/adaptability [82]. - Higher planning success rates (vs. pure rule-based/RL) [110]. - Enhanced training efficiency for RL (rule-based guidance) [110]. - Bridging sim-to-real gap (e.g., LiDAR OGM for consistent representation) [82].	- Complexity of verifying hybrid systems: Interaction between learning and rule-based parts [111]. - Safety of the RL component (black box, OOD). - Interface consistency/robustness: Misinterpretations cause failures. - Balancing control authority between RL and traditional parts. - “Weakest link” problem: Safety depends on both components and interaction.	- Traditional planner: Focus on feasible/efficient paths (precision, efficiency) [109]. - RL agent: Refines for comfort, adapts to dynamics, optimizes further [100]. - Modular assignment simplifies design/tuning.
Hybrid RL Approaches—RL + Model Predictive Control (MPC)	- Integrates RL with MPC. - RL: High-level decisions (strategy, MPC objectives/constraints) [110]. - MPC: Low-level trajectory optimization/control [110]. - Alt: RL learns model for MPC or tunes MPC parameters.	- Optimal low-level control: MPC for constraint-aware execution [110]. - Strategic high-level learning: RL adapts MPC for different scenarios. - Improved trajectory quality and planning time (NN hierarchical DRL + opt. layer) [112].	- Verification complexity of interacting learning/model-based parts [111]. - Safety of RL decision-making component. - Ensuring MPC recursive feasibility given RL goals. - Potential negative interference if RL gives unsuitable objectives to MPC. - “Weakest link” problem applies.	- Hierarchical task decomposition. - RL: Higher-level strategic goals (efficiency, context adaptation). - MPC: Manages precision/comfort for low-level execution (cost function).
Hybrid RL Approaches—DRL + Kinematic-Based Co-pilot	- DRL agent learns primary driving policy [113]. - Kinematic co-pilot: Guidance/constraints for DRL during training (efficiency) and operation (safety/decision support) [113]. - May include rule-based system to assess/mediate final actions for safety [113].	- Enhanced training efficiency for DRL (kinematic guidance) [113]. - Flexible decision-making guidance from co-pilot [113]. - Improved safety/reliability: Rule-based system as safety net [113].	- Verification of interaction (DRL, co-pilot, rule-supervisor) is complex. - Ensuring co-pilot guidance is always safe/beneficial. - Determining override logic for rules (not too conservative/missing DRL insights). - “Weakest link” principle applies.	- Kinematic co-pilot/rules enforce comfort/safety (precision) constraints, guiding DRL [113]. - DRL reward function still primary driver for efficiency/other aspects within safety envelope.

^5^ This table presents a critical evaluation of vehicle control strategies in Autonomous Parking Systems (APSs), synthesized from the information provided in the correspondingly cited references listed within the table (in the ‘Key References’ column). For definitions of abbreviations used, please refer to the ‘Abbreviations’ section at the end of the manuscript. Italicized text or sub-bullets within table cells provide supplementary commentary or specific details.

**Table 6:** Comparative table of pivotal APSs studies and systems ^6^.

Study/System	Core Technological Innovation	Sensor Suite Utilized	AI/ML Methodologies Applied	Key Performance Metrics/Findings	Specific Relevance/Contribution to APS Advancement
RL-OGM-Parking [82]	Hybrid RL (Reeds-Shepp + SAC) planner using LiDAR OGM.	LiDAR	- DRL (SAC) - Rule-based (Reeds-Shepp)	- High PSR (Parking Success Rate) - Reduced ANGS (Number of Gear Shifts) and PL (Path Length) - Outperforms pure rule/learning.	Addresses sim-to-real for learned planners; stable/adaptive maneuvers.
SAC-based DRL for Parking [15]	DRL (SAC) for continuous vehicle control.	LiDAR, Camera	DRL (SAC)	- High PSR - Reduced maneuver times - Robust to dynamic obstacles - Outperforms traditional and other DRL.	Fine-grained control, efficient path gen in dynamic scenarios.
U-Park [67]	User-centric smart parking recommendation for e-micromobility.	Implies Camera (CNN for space detection)	CNN (parking space detection, hazy/foggy)	Tailored recommendations (user pref., conditions).	Extends smart parking to micromobility; user-centric, robust perception.
Diffusion Model Planning [114]	Diffusion models for diverse, feasible motion trajectories.	General AV sensors	Diffusion Models	SOTA PDM score (94.85) on NAVSIM.	Potential for diverse, smooth, context-aware parking trajectories.
4D Imaging Radar for Automotive [62]	4D mmWave radar for high-res point clouds (range, azimuth, elevation, velocity).	4D mmWave Radar	Doppler/angle resolution algos, dynamic CFAR (Constant False Alarm Rate)	Demonstrated 4D high-res imagery in parking lots.	Enhances all-weather perception, velocity measurement for APS.
Monocular VIO (Visual-Inertial Odometry) with Planar Regularities [114]	Monocular VIO regularized by planar features using MSCKF (Multi-State Constraint Kalman Filter).	Monocular Camera, IMU	- MSCKF - Custom Plane Detection	Improved ATE (Absolute Trajectory Error) (1–3 cm accuracy, structured env.).	Precise, cost-effective ego-motion for structured parking (garages).
E2E Parking Dataset and Model) [7]	Open-source dataset (10k+ scenarios) and benchmark for E2E parking.	Multi-camera, Vehicle sensors	- E2E Learning (Transformers) - BEV representation	Baseline: 85.16% success, 0.24 m pos err, 0.34 deg orient err.	Facilitates reproducible research, standardized E2E model benchmarking.
REDFormer [115]	Transformer-based camera-radar fusion for 3D object detection (low-visibility).	Camera, Radar (nuScenes)	- Transformers - BEV Fusion	Improved perf. in rain/night on nuScenes.	Enhances APS perception robustness in adverse conditions.
Improved CILQR (Constrained Iterative Linear Quadratic Regulator) [116]	Enhanced Constrained Iterative LQR for stable, human-like, efficient trajectory planning.	Assumed std. AV sensors	- CILQR - Hybrid A*	Improved human-like driving, traffic efficiency, real-time capability.	Potential for higher quality, smoother, natural parking trajectories.
AVP HMI (Human–Machine Interface) Explanations [117]	Scenario-based XAI framework for AVP HMI.	N/A (HMI study)	XAI principles	Improved driver trust and UX; reduced mental workload; better user perf.	Improves user understanding, trust, acceptance of AVP via transparency.
Hyundai Mobis Parking System [118]	Commercial Advanced Automated Parking System (AAPS).	Ultrasonic, Surround-view cameras	Proprietary map gen and autonomous parking algos	Seamless autonomous parking (single touch).	Real-world deployment, commercialization of in-vehicle APS tech.

^6^ This table presents a comparative overview of pivotal Autonomous Parking Systems (APSs) studies and systems, synthesized from the information provided in the correspondingly cited references listed within the table (in the ‘Study/System column). For definitions of abbreviations used, please refer to the ‘Abbreviations’ section at the end of the manuscript. Italicized text or sub-bullets within table cells, if any, provide supplementary commentary or specific details.

## Abbreviations

The following abbreviations are used in this manuscript:

AAPS	Advanced Automated Parking System
ADAS	Advanced Driver Assistance Systems
AI	Artificial Intelligence
ANGS	Number of Gear Shifts
APA	Autonomous Parking Assist
APF	Artificial Potential Field
APS	Autonomous Parking Systems
ATE	Absolute Trajectory Error
AV	Autonomous Vehicle
AVP	Autonomous Valet Parking
BEV	Bird’s Eye View
Bi-RRT*	Bidirectional Rapidly exploring Random Tree*
CAN	Controller Area Network
CAV	Connected Automated Vehicles
CFAR	Constant False Alarm Rate
CILQR	Constrained Iterative Linear Quadratic Regulator
CMDP	Constraint-driven Markov Decision Process
CMOS	Complementary Metal-Oxide Semiconductor
CNN	Convolutional Neural Networks
CP	Control Points
CV	Computer Vision
DAVIS	Dynamic and Active Pixel Vision Sensor
DL	Deep Learning
DRL	Deep Reinforcement Learning
DVS	Dynamic Vision Sensor
ECU	Electronic Control Unit
EKF	Extended Kalman Filter
EMPC	Economic Model Predictive Control
E2E	End-to-End
FAST-LIO	Fast LiDAR-Inertial Odometry
FoV	Field-of-View
FPGA	Field-Programmable Gate Array
FPS	Frames Per Second
GA	Genetic Algorithm
GF-LIO	Global Factor-based LiDAR-Inertial Odometry
GP	Gaussian Process
GPS	Global Positioning System
GPU	Graphics Processing Unit
HDR	High Dynamic Range
HER	Hindsight Experience Replay
HMI	Human–Machine Interface
HMRP	Human-Manipulated Risk Perception
ICBS	Improved Conflict-Based Search
IDS	Intrusion Detection Systems
IMU	Inertial Measurement Unit
IR	Infrared
I-STC	Improved Safe Travel Corridor
LIO-SAM	LiDAR-Inertial Odometry via Smoothing and Mapping
LKF	Linear Kalman Filter
LPV	Linear Parameter Varying
LSS	Lift Scene Splatting
LWIR	Long-Wave Infrared
mAP	mean Average Precision
MBSE	Model-Based Systems Engineering
MEMS	Micro-Electro-Mechanical Systems
ML	Machine Learning
MORL	Multi-Objective Reinforcement Learning
MPC	Model Predictive Control
MPS	Mobis Parking System
MSCKF	Multi-State Constraint Kalman Filter
MTBF	Mean Time Between Failures
NIR	Near-Infrared
NMPC	Nonlinear Model Predictive Control
NN	Neural Network
OCP	Optimal Control Problem
ODD	Operational Design Domain
OGM	Occupancy Grid Map
OPA	Optical Phased Array
PDM	Probabilistic Diffusion Model
PID	Proportional Integral Derivative
PINS	Pontryagin’s Indirect Method Solver
PL	Path Lenght
PNN	Pseudo-Neural Network
PSR	Parking Success Rate
QP	Quadratic Programming
ReLU	Rectified Linear Unit
RGB	Red
RL	Reinforcement Learning
RPROP	Resilient Propagation
RS	Reeds-Shepp
RRT	Rapidly exploring Random Tree
RRT*	Rapidly exploring Random Tree Star
RRT-Connect	Rapidly exploring Random Tree Connect
SAC	Soft Actor-Critic
SLAM	Simultaneous Localization and Mapping
SMC	Satisfiability Modulo Convex
SNR	Signal-to-Noise Ratio
SoC	System-on-Chip
SOTA	State-Of-The-Art
SOTIF	Safety Of The Intended Functionality
SQP	Sequential Quadratic Programming
SSL	Solid-State LiDAR
SWIR	Short-Wave Infrared
T-ITS	IEEE Transactions on Intelligent Transportation Systems
T-IV	IEEE Transactions on Intelligent Vehicles
UGV	Unmanned Ground Vehicle
UKF	Unscented Kalman Filter
UX	User Experience
V&V	Verification and Validation
V2X	Vehicle-to-Everything
V2I	Vehicle-to-infrastructure
VIO	Visual-Inertial Odometry
XAI	Explainable AI
YOLO	You Only Look Once
